# Immunogenetic variation shapes the gut microbiome in a natural vertebrate population

**DOI:** 10.1186/s40168-022-01233-y

**Published:** 2022-03-08

**Authors:** Charli S. Davies, Sarah F. Worsley, Kathryn H. Maher, Jan Komdeur, Terry Burke, Hannah L. Dugdale, David S. Richardson

**Affiliations:** 1 School of Biological Sciences, University of East Anglia, Norwich Research Park, Norfolk, NR4 7TJ UK; 2 NERC Biomolecular Analysis Facility, Department of Animal and Plant Sciences, University of Sheffield, Sheffield, S10 2TN UK; 3 Groningen Institute for Evolutionary Life Sciences (GELIFES), University of Groningen, P.O. Box 11103, 9700 CC Groningen, The Netherlands; 4 Faculty of Biological Sciences, School of Biology, University of Leeds, Leeds, LS2 9JT UK; 5 Nature Seychelles, Roche Caiman, Mahé, Republic of Seychelles

**Keywords:** Major Histocompatibility Complex, Gut microbiome, Microbial diversity, *Acrocephalus sechellensis*, Genetic variation, Life history

## Abstract

**Background:**

The gut microbiome (GM) can influence many biological processes in the host, impacting its health and survival, but the GM can also be influenced by the host’s traits. In vertebrates, Major Histocompatibility Complex (MHC) genes play a pivotal role in combatting pathogens and are thought to shape the host’s GM. Despite this—and the documented importance of both GM and MHC variation to individual fitness—few studies have investigated the association between the GM and MHC in the wild.

**Results:**

We characterised MHC class I (MHC-I), MHC class II (MHC-II) and GM variation in individuals within a natural population of the Seychelles warbler (*Acrocephalus sechellensis*). We determined how the diversity and composition of the GM varied with MHC characteristics, in addition to environmental factors and other host traits. Our results show that the presence of specific MHC alleles, but not MHC diversity, influences both the diversity and composition of the GM in this population. MHC-I alleles, rather than MHC-II alleles, had the greatest impact on the GM. GM diversity was negatively associated with the presence of three MHC-I alleles (*Ase-ua3, Ase-ua4, Ase-ua5*), and one MHC-II allele (*Ase-dab4*), while changes in GM composition were associated with the presence of four different MHC-I alleles (*Ase-ua1, Ase-ua7, Ase-ua10, Ase-ua11*). There were no associations between GM diversity and *TLR3* genotype, but GM diversity was positively correlated with genome-wide heterozygosity and varied with host age and field period.

**Conclusions:**

These results suggest that components of the host’s immune system play a role in shaping the GM of wild animals. Host genotype—specifically MHC-I and to a lesser degree MHC-II variation—can modulate the GM, although whether this occurs directly, or indirectly through effects on host health, is unclear. Importantly, if immune genes can regulate host health through modulation of the microbiome, then it is plausible that the microbiome could also influence selection on immune genes. As such, host–microbiome coevolution may play a role in maintaining functional immunogenetic variation within natural vertebrate populations.

**Video abstract**

**Supplementary Information:**

The online version contains supplementary material available at 10.1186/s40168-022-01233-y.

## Background

Most animals harbour a complex microbial community including bacteria, archaea, viruses, and microbial eukaryotes, collectively known as the host’s microbiome. Members of this diverse community have coevolved with their host’s over evolutionary time and can play a fundamental role in their host’s biology [1]. Consequently, it is important to understand the ecological and evolutionary processes that shape host-associated microbial communities [2]. This is particularly true of the vertebrate gut, where the gut microbiome (GM) contributes to many key biological processes in the host, from enabling nutrient uptake [3] to pathogen defence [4]. As such, studies on domestic or laboratory animals have demonstrated that disrupting the GM can have negative consequences for host health and survival [5]. However, the factors that govern the diversity and composition of the GM remain unclear, particularly in natural populations where organisms are exposed to greater levels of environmental and microbial complexity compared to domestic or laboratory animals [6].

Studies on wild vertebrates are now attempting to unravel the importance of extrinsic factors, such as the environment, season and diet in shaping GM variation among species, populations, and individuals e.g. [7–9]. Within a population, individual variation in the GM has also been linked to a multitude of host factors including sex [10], age [11], body condition [12], cognition [13], sociality [14], and hormones [15, 16]. Host genetics may also play an important role in determining individual GM variation within a population [17]. For example, increased genetic relatedness predicts decreased GM dissimilarity [10, 18] and compositional changes [19, 20].

Immunogenetic variation may play an important role in driving individual differences in the GM (reviewed in [21]). Microbial complexity presents a unique challenge to the host immune system, which has evolved to prevent pathogenic microbes from proliferating, while still enabling beneficial microbes (i.e., those that form mutualistic/commensal interactions) to remain [22, 23]. In humans, inappropriate immune responses can lead to detrimental compositional changes to the GM via a loss of diversity and stability, as well as an increase in the proliferation of pathogenic bacteria [24]. Such changes in GM composition can lead to serious health outcomes [5, 25], so accurate regulation of microbes by the host is essential. Variation in GM composition has been associated with variation in immune receptor genes (which detect antigens, inducing an immune response) [26], making them key candidate genes that might influence the relationship between host genotype and the GM. For example, pattern recognition receptor genes involved in the innate immune response, including Toll-like receptors (TLR), nucleotide-binding oligomerization-like receptors, RIG-I-like receptors and C-type lectin receptors, play an important role in recognising and regulating the GM in humans and captive animals (reviewed in [26, 27]).

In vertebrates, Major Histocompatibility Complex (MHC) genes, which play a key role in antigen detection in the adaptive immune response [28], may also shape GM variation. A specialised group of host cell-surface glycoproteins encoded by MHC genes, recognise and bind protein antigens (including microbial derived peptides), presenting them to T lymphocytes and B cell receptors. If the combined MHC-peptide complex is recognised as non-self, the T and B cells are activated, triggering an appropriate immune cascade [29]. After such a reaction, the adaptive immune system produces memory cells, enhancing any future immune response (an acquired response) if the host re-encounters that specific antigen. MHC class I (MHC-I) molecules, which are expressed on the surface of virtually all somatic cells, primarily present intracellular peptides to cytotoxic CD8^+^ T cells, while MHC class II (MHC-II) molecules are present on antigen-presenting cells and mainly present extracellular antigens to helper CD4^+^ T cells, including those from bacterial species in the intestinal tract [30, 31]. Functional variants of MHC genes can confer differential antigen recognition and affect fitness [32, 33], with pathogen-mediated selection thought to drive the extraordinary within- and among-population variation observed at the MHC [34, 35]. Likewise, individuals with different MHC genotypes are likely to respond differently to different microbial species, including commensals in the gut [36], which could contribute to the inter-individual GM variation seen in natural vertebrate systems. Comparing functional immunogenetic variation with the diversity and composition of the GM could provide further understanding of the selective pressures acting on the maintenance of host genetic variation, with the GM being one possible factor driving selection at MHC genes.

Various studies on captive animals have found links between individual MHC variation and GM composition [37–39]. However, captivity can profoundly alter an organism’s microbiome [40] and the complexities associated with natural populations cannot be captured in such studies [6]. Few studies have investigated associations between the microbiome and individual MHC variation in wild animals. Those that have, have primarily focused on MHC-II variation, as this is central to humoral immunity against extracellular microbes [41]. For example, in the reddish-grey mouse lemur (*Microcebus griseorufus),* the presence of specific MHC supertypes was associated with changes in GM composition, but not diversity [42]. In threespine sticklebacks (*Gasterosteus aculeatus*), GM diversity and MHC-II diversity were negatively correlated, and changes in GM composition and diversity were associated with specific MHC-II alleles [43]. In contrast, in two species of giant salamander, the Ozark hellbender (*Cryptobranchus alleganiensis bishopi*) and eastern hellbender (*C. a. alleganiensis*), although the composition of the cutaneous microbiome was associated with specific MHC-II alleles, cutaneous microbiome diversity and composition were positively correlated with individual MHC-II divergence [44]. Lastly, studies on Leach’s storm petrel (*Oceanodroma leucorhoa*), and the blue petrel (*Halobaena caerulea*), found that variation in body surface (feather and glandular) bacterial communities, responsible for the production of volatile cues involved in mate choice, was associated with MHC-II variation [45, 46]. However, very few studies have tested for an association between MHC-I genes (or any other host immune genes) and the microbiome in a natural population (but see [19, 42]). This is despite the fact that MHC-I variation could impact the GM indirectly, for example via differences in individual susceptibility to intracellular infections (such as viruses) that could then impact the health and/or GM of the host [47].

The isolated population of Seychelles warblers (*Acrocephalus sechellensis*) on Cousin Island has been intensively monitored since 1985 [48, 49]—and represents an excellent study system in which to investigate associations between MHC variation and the GM in the wild. The age, sex, status and territory of nearly every individual is known, and DNA samples have been collected since 1990 [50]. This population harbours reduced genetic variation as a result of a past genetic bottleneck [51]. However, variation still exists across the genome [52] and, importantly, at both MHC-I [53] and MHC-II [54] loci, as well as at some TLR genes [55]. Differences in individual fitness have been linked to this genetic variation, for example, individual condition [56] and reproductive success [57] are negatively correlated with genome-wide homozygosity. Likewise, differential survival and reproductive success are associated with variation in the viral-sensing *TLR3* gene [50]. Lastly, survival is positively associated with a specific MHC-I allele (*Ase-ua4)* and MHC-I diversity [58], and the occurrence of extra-pair paternity is negatively related to MHC-I diversity of the social male [59]. But whether there is functional variation at MHC-II in the Seychelles warbler and if this is related to variation in fitness remains unresolved.

Here, we aim to test whether immunogenetic variation, alongside other host and environmental factors, is associated with individual GM variation in the Seychelles warbler. Specifically, we test if bacterial diversity and community composition are associated with MHC-I and MHC-II gene diversity, or the presence of specific alleles at MHC-I, MHC-II, or *TLR3* loci. It is difficult to make clear predictions about such associations. Individual GM diversity might be negatively associated with immunogenetic variation [43] if this genetic diversity enables hosts to recognise and instigate an immune response against more bacterial species. Alternatively, GM diversity might be positively associated with greater, or optimal, immunogenetic diversity. This could occur via two different pathways: first, directly, with greater immunogenetic diversity helping to eliminate highly competitive (potentially pathogenic) bacterial taxa, while still tolerating a network of commensal/mutualistic bacterial species—for example, there is evidence that following peptide-binding, the MHC can also enable tolerance towards certain microbial species including gut commensals; this can be induced through mechanisms involving regulatory T cells or immunoglobulin A [60–62]- and second, indirectly, with greater, or optimal, immunogenetic diversity conferring increased host health and fitness, which in turn may be associated with greater GM diversity [24]. We also predict that GM composition and diversity will differ in relation to the presence or absence of specific immune alleles via differences in immunity and tolerance. This is expected to be most marked for MHC-II alleles, as these are expressed extracellularly on antigen-presenting cells that can extend into the gut lumen and are therefore important in the recognition of gut microbes [22].

## Methods

### Study species and sample collection

The Seychelles warbler is a small insectivorous passerine, endemic to the Seychelles archipelago. They are facultative cooperative breeders, defending strict year-round territories [48]. The population on Cousin Island (4°20’S,55°40’E; 0.29 km^2^)—which has been extensively monitored since 1985 [48, 49] has a carrying capacity of ca. 320 adult individuals, existing in ca. 115 territories [48, 63, 64]. There is virtually no migration to or from Cousin [65]. Individuals can live for a maximum of 19 years, with a median post-fledging lifespan of 5.5 years [66]. A comprehensive population census is conducted bi-yearly during the major breeding season (June–September) in the south-east monsoon, and the minor breeding season (January–March) in the north-west monsoon [67]. Territory quality (as defined by insect prey availability) varies quantifiably within and between years [68]; thus, it is possible to separate the influence of environmental factors from host-intrinsic factors when investigating individual GM variation.

Individuals are either caught as chicks in the nest or by mist net. Each bird is given a metal British Trust for Ornithology (BTO) ring and a unique combination of three colour rings, allowing it to be individually identifiable. Birds are aged based on hatch date, behaviour, or eye colour; grey eyes indicate an age < 5 months, light brown eyes are characteristic of sub-adults (5–12 months), and adults (> 12 months) have red-brown eyes [48, 69]. Blood samples (25 μl) are collected by brachial venipuncture and stored in 0.8 ml of absolute ethanol at either room temperature or 4°C.

In total, 343 faecal samples were collected from 293 captures, across two years and three consecutive field seasons: the major 2017, minor 2018, and major 2018 breeding seasons. Captured birds were immediately placed into a clean bag. In the first major breeding season of our study (2017), this was a laundered cotton bag; however, for all following seasons, birds were placed into a single use plastic-lined, flat-bottomed paper bag containing a plastic tray covered by a metal grate, according to an established protocol [70]. The metal grate and tray were sterilised with a 10% bleach solution between use. Individuals were removed from the bag once they had defaecated or after a maximum of 30 min. A sterile flocked swab was used to transfer faecal material into a sterile microcentrifuge tube containing 1 ml of absolute ethanol. If the bird defaecated outside of the bag or tray, then a sample was still collected. Control samples from possible sources of contamination such as the bag, grate, and tray were taken throughout each sampling season, using sterile flocked swabs (*n* = 1 per type). In addition to this, four swab samples were taken from fieldworkers’ hands throughout each sampling season; the gDNA from different hand samples was pooled into one sample prior to sequencing. Faecal samples were stored in the field at 4°C for a maximum of 3 months (mean days ± SEM: 38.1 ± 1.3), before being transported to the lab, where they were stored at −80°C prior to extraction. There was no association between faecal GM diversity (measured as Shannon, Chao1 and Faith's phylogenetic diversity) or composition (measured as weighted UniFrac), and extraction time (time in days from collection to extraction) or sequencing run (*n* = 195, *P* > 0.05).

### Molecular methods

Genomic DNA (gDNA) was extracted from blood using the DNeasy blood and tissue kit (Qiagen). Individuals were genotyped at 30 polymorphic microsatellite loci [52, 64], and standardised individual microsatellite heterozygosity (*H*_s_) was calculated using the R 3.6.1 function Genhet 3.1 [71]. Sex was determined via PCR [64, 72]. Variation at one non-synonymous SNP within the leucine-rich repeat domain of *TLR3* exon 4 was determined following [50].

#### MHC sequencing and bioinformatics

In total, 314 samples were MHC sequenced, including 229 samples from individuals that had GM data and 31 samples from individuals previously MHC screened at either MHC-I [73] or MHC-II [54] using older techniques. The remainder included 30 repeated samples, 23 negative controls (making up at least 5% of each plate) and four samples (one per plate) from one great reed warbler (*Acrocephalus arundinaceus*) individual to serve as a positive control.

MHC-I exon 3 and MHC-II exon 2 were amplified and sequenced using previously validated primer sets [48, 49] (Additional file 1: Table S1), with the addition of Illumina index sites. Additionally, six random hexamers (N) were added to the first round PCR (PCR1) primers to increase diversity and improve cluster separation [74]. The two primer pairs amplifying MHC-I each included a motif-specific primer situated within exon 3, and one general primer situated in intron 3, and so amplified 262 bp of the full exon (274 bp). These primers had been designed to preferentially amplify functional variants, while avoiding known pseudogenes [53, 75]. The primers for MHC-II, situated within the flanking introns 1 and 2 of exon 2, amplify a 291-bp fragment. These sequences were then edited to the 270 bp MHC-II exon 2 [54]. The term ‘allele’ is used to describe the different variants amplified for each class of MHC, consistent with other publications investigating MHC diversity; however, alleles cannot be assigned to specific (duplicated) loci within each MHC class. Previous work suggests that, in the Seychelles warbler, there are a minimum of four duplicated MHC-I loci and six MHC-II loci [53, 54]. Sequencing of the MHC-I and MHC-II exons was carried out using 2x 250-bp paired-end sequencing on an Illumina MiSeq platform (Illumina, San Diego) (see Additional file 1: Supplementary methods for details).

Processing and MHC genotyping of raw Illumina MiSeq data were conducted using the Amplicon Sequencing Assignment (AmpliSAS) tool [76]. First, FastQC was used to check read quality, before merging pair-ended reads together using AmpliMERGE (10,257,832 merged sequences). AmpliCLEAN was then used to remove low-quality reads (Phred score of <30) and any that were missing either primers or barcodes (e.g., from residual PhiX). MHC-I and MHC-II sequences were separated resulting in 3,044,897 raw MHC-I sequences and 6,144,575 raw MHC-II sequences. All downstream bioinformatics and analysis were conducted separately for MHC-I and MHC-II. Cutadapt 1.6 [77] was used to remove MHC-specific primers, the six random hexamers, and short reads (<100 bp). For MHC-II sequences, remaining intron regions were also removed, leaving a 270-bp fragment spanning the full exon. AmpliCHECK was used for preliminary data exploration, using Illumina-based default settings. Finally, AmpliSAS was used for demultiplexing, clustering, and filtering reads. First, a subset of 30 duplicated samples were used to optimise parameters for MHC-I and MHC-II, testing both minimum dominant frequency settings for the clustering step, and minimum amplicon depth for the filtering step, as recommended in [76]. Based on these results (Additional file 1: Table S2, S3) Illumina default clustering settings were used (1% substitution errors, 0.001% indel errors, 25% minimum dominant frequency) for both MHC classes. For the filtering step, chimaeras and sequences that only appeared in one sample were removed, and the minimum amplicon frequency was set as 1.6% for MHC-I and 1.8% for MHC-II. This resulted in 1,267,410 raw MHC-I sequences and 1,385,049 raw MHC-II sequences. Due to computational limitations, the MHC-II dataset was split into two halves and analysed using AmpliSAS separately, before being combined using AmpliCOMBINE in the web version of AmpliSAS.

For MHC-I, the majority of putative alleles were 262 bp, although three sequences that were <262 bp were present in >80% individuals. These were not homologous to any known MHC gene when checked using blastn and were removed from downstream analysis. The majority of MHC-II putative alleles were the full 270 bp length, although there were also sequences between 267 and 269 bp, which were similar to MHC genes (see results). All MHC-II sequences <267 bp in length were not similar to any known MHC genes and, as putative sequencing artefacts, were removed from downstream analysis. MHC-I and MHC-II putative alleles were first checked against all known Seychelles warbler alleles. Any unknown putative alleles were then checked against the GenBank (NCBI) nucleotide database (accessed on June 25, 2020) to assess similarity to known MHC alleles from other related species. Additionally, samples of insufficient read depth based on rarefaction curves, which equated to a minimum read depth of 150 per amplicon for MHC-I, and 100 per amplicon for MHC-II, were removed. For 30 individuals sequenced twice to confirm repeatability, the sample with the greatest read depth was retained. After processing, the total number of reads assigned to an allele was 1,071,525 for MHC-I (mean ± SEM = 4391.5 ± 149.3 per sample) and 1,123,211 for MHC-II (mean ± SEM = 4603.3 ± 888.2 per sample) in the Seychelles warbler.

#### Microbial extraction, sequencing, and bioinformatics

In total, 400 faecal samples were sequenced across three sequencing runs (two plates per sequencing run). This included 343 unique faecal samples (from 235 individuals) and 14 control samples, the latter of which included six extraction negative controls, four positive controls (using a microbial community standard), and four sampling controls. Additionally, 43 faecal samples were sequenced twice (20 within the same run and 23 across different runs) to determine sequencing accuracy and repeatability within, and between runs.

Faecal samples were centrifuged for 10 min at 10,000 rpm, and the supernatant was removed. To remove any residual ethanol, the resulting pellet was washed with 100 μl RNase/DNA-free molecular grade water by centrifuging at 10,000 rpm for 10 min. The supernatant was then removed, and the washing step repeated a further two times. Microbial DNA was extracted from 0.05–0.1 g of each sample using the DNeasy PowerSoil Kit (Qiagen), according to an optimised version of the manufacturer’s instructions. Modifications consisted of a heat block step (65°C for 10 min) prior to bead-beating and elution of DNA in a final volume of 60 μl elution buffer. To further assess sequencing accuracy and repeatability between runs, a ZymoBIOMICS microbial community standard (D6300) was extracted as a positive control using a ZymoBIOMICS DNA miniprep kit (Zymo Research), according to the manufacturer’s instructions. The observed microbiome profiles of the positive control represented the expected microbial community (8 ASVs). The accuracy and repeatability of the DNeasy PowerSoil Kit extraction method were assessed and validated in a separate study which also utilised the ZymoBIOMICS microbial community standard (D6300).

Extracted gDNA was quantified using a Qubit 4.0 Fluorometer (Invitrogen) with a Qubit dsDNA HS assay kit (Invitrogen). Aliquots of gDNA were shipped on dry ice to the Centre for Genomic Research, University of Liverpool, for library preparation, pooling and sequencing. Bacterial barcoding was performed with a 2-step amplification process using the primers 515F (5'TGCCAGCMGCCGCGGTAA3’) and 806R (5’GGACTACHVGGGTWTCTAAT3’) [78], which amplify the V4 region of the 16S rRNA gene (see Additional file 1: Supplementary methods for details). Paired-end sequencing was carried out using 2x 250-bp paired-end sequencing on an Illumina MiSeq platform (Illumina, San Diego).

For each sequencing run, raw reads were first trimmed using Cutadapt 1.2.1 [77] to remove Illumina adapter sequences. Reads were further trimmed using Sickle 1.200 with a minimum window quality score of 20, resulting in 12,308,047, 9,397,303 and 9,831,508 demultiplexed reads for the three runs, respectively (mean ± SEM per sample: 102,567.1 ± 10,454.8, 67,123.6 ± 6,633.1, 70,225.1 ± 5,423.5). Sequences were then analysed using QIIME2 2019.10 [79]. Based on overall quality scores, the first 10 bases of each read were trimmed, and sequences truncated to 210 bp for both forward and reverse reads. The DADA2 plugin 2019.10.0 was used to join paired-end reads, denoise, remove chimaeras and residual PhiX reads, dereplicate and call amplicon sequence variants (ASVs) [80, 81]. Following this, results from the three separate runs were merged, resulting in a total of 22,997,693 reads (mean ± SEM per sample: 57,494.3 ± 3424.8) with 36,182 ASVs. A mid-point rooted phylogeny was then constructed using the masked alignment MAFFT [82] and the Fast Tree approach [83]. Taxonomic assignment of ASVs was performed by training a naïve-Bayes classifier on the SILVA 132 16S dataset using 99% sequence similarity [84, 85]. Plastid-like and archaeal sequences were removed, as well as amplicons which only had one read across the whole dataset (singletons) which likely represent sequencing errors. In total, ASVs from 19 genera were present in the negative control samples (Additional file 2). Of these, 15 genera were present at < 3% overall abundance in the negative control samples (and had greater read counts in the faecal samples) and so were not considered as contaminants introduced at extraction. Furthermore, two genera had fewer than 500 reads across faecal samples, and one genus was found at equal abundance in negative extraction controls and faecal samples from different sequencing runs, and so it could not be determined which sample was contaminated; as such, these sequences were retained in analysis. A visual assessment of samples showed that two ASVs were present at relatively high sequencing depth in negative extraction controls—these were also prevalent in faecal samples with low sequencing depth and DNA concentration and so were removed as probable contaminants. These two ASVs were additionally confirmed as contaminants using the prevalence and filtering methods in the DECONTAM package [86]. One of these ASVs was from the genus *Defltia* (relative abundance of 90.5% in a negative extraction control from the first run), and one was from the genus *Limnobacter* (relative abundance of 99.9% in a negative extraction control from the third run). The removal of these ASVs resulted in a total of 21,863,215 reads (mean ± SEM per sample: 54,795.0 ± 3,439.6) with 34,869 ASVs. Following cleaning, visual assessment of the positive controls revealed that only the eight expected ASVs were present, while visual assessment of the collection controls showed they had quite different bacterial profiles when compared to the faecal samples, with few overlapping ASVs at high abundance. This indicates that neither the sequencing nor the sampling methods significantly impacted taxa present in the faecal samples. The final sample metadata, ASV and taxonomy tables were all exported from QIIME2 into R 3.6.1 where they were processed using phyloseq 1.28.0 [87]. Sample completeness and rarefaction curves were generated using iNEXT 2.0.20 [88]; completeness plateaued at approximately 10,000 reads and 34 samples (including all six negative extraction controls) with fewer reads were excluded from downstream analyses (Additional file 1: Fig S1). Overall, 320 unique samples (93%) were retained from 224 individuals.

The repeatability of GM sequencing was tested by comparing samples that were sequenced multiply within and across sequencing runs and that had sequencing depth of >10,000 reads (37 out of the initial 43 samples). Individual repeatability was tested for individuals that had multiple samples collected from the same capture or were caught multiple times in the same field season and that had a sequencing depth of >10,000 reads (115 samples from 51 individuals). The individual repeatability dataset was further filtered to remove ASVs that had a total read count of <50 across samples and could potentially represent sequencing errors. Euclidean dissimilarity between pairs of samples was compared using one metric of alpha diversity (the Shannon diversity index) and two metrics of beta diversity (unweighted and weighted UniFrac of between and within duplicated samples) using Kruskal–Wallis tests.

### Statistical analyses

Unless otherwise stated, all analyses were conducted in R 3.6.1. To characterise the Seychelles warbler GM, samples sequenced twice for repeatability analysis (sample duplicates) were filtered such that only the sample with the greatest read-depth was retained. Samples collected from the same bird during the same catch session (catch duplicates) were filtered to retain the single sample with the smallest potential exposure to external contamination, i.e., samples collected from cleaned trays were prioritised over those collected from other substrates, then the sample with the highest read depth was prioritised. The removal of sample and catch duplicates resulted in 281 samples (from 224 individuals). This resulted in a total of 16,562,592 reads (mean ± SEM per sample: 58,941.6 ± 3997.7) with 34,869 ASVs in the faecal dataset. For all alpha diversity, beta diversity and differential abundance analyses, microbiome samples taken from chicks were excluded due to a small sample size (*n* = 11). Individuals with incomplete MHC genotype data (*n* = 25) were also excluded. Lastly, to prevent pseudo-replication, where an individual had multiple samples taken at different capture events, a single sample was selected at random, giving an overall sample size of 195 samples from 195 individuals from Cousin Island. Prior to downstream analysis the dataset was further filtered to remove ASVs that had a total read count of <50 across samples and could potentially represent sequencing errors. This resulted in a total of 10,680,281 reads (mean ± SEM per sample: 54,770.7 ± 4,170.4) with 9,628 ASVs in the cleaned, non-rarefied dataset.

#### Alpha diversity

All 195 samples were rarefied to a depth of 10,000 reads, based on sample completeness curves, leaving a total of 1,950,000 reads and 27,547 ASVs across samples in the rarefied dataset. Analyses were run using both rarefied and non-rarefied data; however, as results were comparable between datasets and library size was highly variable across samples, only the outcome of models using the rarefied dataset are presented. Three metrics of alpha diversity were calculated: Chao1 [89] (microbial species richness), Shannon diversity index [90] (species richness, taking into account sample evenness) and Faith’s phylogenetic diversity index [91] (the phylogenetic diversity of a sample). Chao1 and Shannon diversity indices were calculated using phyloseq 1.28.0 [87], and Faith’s phylogenetic diversity was calculated using btools 0.0.1 [92]. Both Chao1 and Faith’s phylogenetic diversity were log-transformed in models to improve residual fit.

Linear models with a Gaussian distribution were constructed using glmmTMB 0.2.3 [93] to determine whether the alpha diversity of the GM differed with (1) the presence/absence of individual immune genes and (2) immune gene diversity. For each diversity metric two models were run. The first model included the presence/absence of all the MHC-I and MHC-II alleles that were present in at least 15% of sampled individuals and that were the correct length (see above). This included *Ase-dab3, Ase-dab4, Ase-dab5, Ase-ua1/10, Ase-ua3, Ase-ua4, Ase-ua5, Ase-ua6, Ase-ua7, Ase-ua8, Ase-ua9,* and *Ase-ua11 (Ase-ua1* and *Ase-ua10* were perfectly correlated, so only *Ase-ua1* was included). The second model contained MHC-I diversity, MHC-II diversity, and the squares of each of these terms, since optimal, rather than a greater diversity, of MHC alleles could be more beneficial [94]. Both models also included individual heterozygosity (*H*_s_) and *TLR3* genotype (*TLR3*^*AA*^, *TLR3*^*AC*^ or *TLR3*^*CC*^). The field period sampled (major 2017, major 2018, minor 2018), sex (male, female), and age (fledgling, old fledging, sub-adult, adult) were also included, as these factors have been shown to influence GM variation in other studies. Alpha diversity (Shannon, log Chao1, log Faith) was entered as the response variable. In all models, continuous factors were standardised (scaled and centred) using arm 1.10-1 [95]. Biologically relevant interactions were initially included in models but were removed prior to model averaging to enable interpretation of first-order effects, as all were non-significant (*P* < 0.1). Field period and territory quality were correlated (linear model; *F*_2,185_ = 117.2, *P* < 0.001), so only field period was included in the models. Collinearity between independent variables was tested using variance inflation factors ensuring an upper limit of three. Collinearity between the presence/absence of immune alleles was further assessed using GGally 2.0.0 [96]. DHARMA 0.2.4 [97] was used to confirm that there was no over or under dispersion, or residual spatial or temporal autocorrelation in the models. Model averaging—an information-theoretic approach using the dredge function in MUMIn 1.43.6 [98]—was used to select plausible models. All models within seven AICc of the top model were included in the averaged model, to obtain the final conditional model [99].

#### Beta diversity

The unrarefied dataset was further filtered to remove ASVs that appeared in fewer than five samples, based on an assessment of overall ASV prevalence and abundance (Additional file 1: Fig S2). Overall, 1,934 ASVs were retained following filtering. To account for uneven sequencing depth across samples, reads were normalised using the cumulative sum scaling function [100] in metagenomeSeq 1.26.3 [101]. This method includes adding a pseudocount and transforming the data (log (x + 0.0001). Therefore, to preserve zeros from the original counts, post-transformation values were corrected by subtracting the log of the pseudocount [102]. Two beta diversity metrics that incorporate phylogenetic distance were then calculated using phyloseq 1.28.0 [103]; these were unweighted UniFrac distance (based on the presence/absence of microbial taxa) and weighted UniFrac distance (a quantitative measure which accounts for differences in the abundances of microbial taxa) [104]. Marginal Permutational Analysis of Variance tests (PERMANOVAs) were used to assess whether GM community composition differed with immune gene characteristics, using the adonis2 function in Vegan 2.5.6 [105] with 10,000 permutations. As with alpha diversity models, two sets of PERMANOVA tests were constructed for each beta diversity metric, with the first set of models including the presence/absence of MHC alleles, and the second set of models including MHC diversity; other variables were included as in alpha diversity models. To clarify whether significant differences detected in PERMANOVA tests were caused by differences in mean values, rather than variation in dispersion across groups [106], homogeneity of group dispersions was tested using the betadisper function in Vegan 2.5.6 [105]. Principle coordinate analysis (PCoA), based on weighted and unweighted UniFrac distances, was used to visualise the differences in composition between groups.

#### Differential abundance analysis

To assess whether particular ASVs were differentially abundant between groups of individuals with different immune gene characteristics, DESeq2 1.24.0 [107] was used. For this analysis, unrarefied reads were filtered following the same protocol as used for beta diversity analysis, but untransformed, as DeSeq2 uses its own variance stabilising transformation to account for variation in library size across samples. Overall, 1,934 ASVs were retained following filtering. DeSeq2 estimates the log2-fold change in microbial abundance between sample groups using a negative binomial distribution to model ASV counts. Only variables that were associated with significant compositional shifts in PERMANOVA tests (*Ase-ua7, Ase-ua11, Ase-ua1/10,* field period, and age class) were included in this analysis to avoid over-parametrization (Table 1). To account for the large number of zero counts for individual ASVs, the “poscounts” estimator was included when estimating size factors. As part of the DeSeq2 pipeline, differential ASV abundance was assessed using negative binomial Wald tests and *P* values were adjusted using the Benjamini and Hochberg false-discovery rate correction, with a significance cut-off of *P* < 0.01. Two ASVs did not converge due to a high number of zero counts across samples; these were removed from the analysis.

**Table 1:**
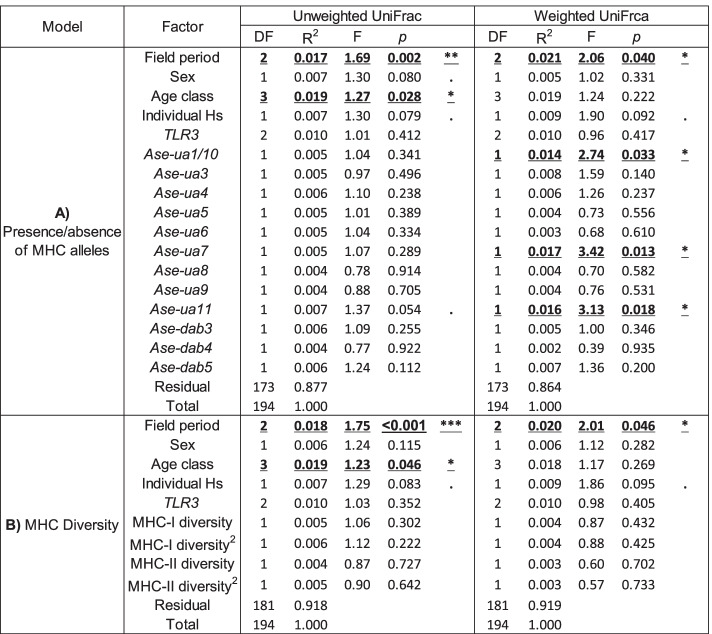
The effect of host-associated variables on gut microbiome composition in the Seychelles warbler (*n* = 195). Unweighted and weighted UniFrac were used as beta diversity metrics in separate PERMANOVA models. **A** Including the presence/absence of MHC alleles or **B** MHC diversity. Significant terms are in bold and underlined. **P < 0.01 and *P < 0.05

## Results

### Seychelles warbler GM profile

**Fig. 1:**
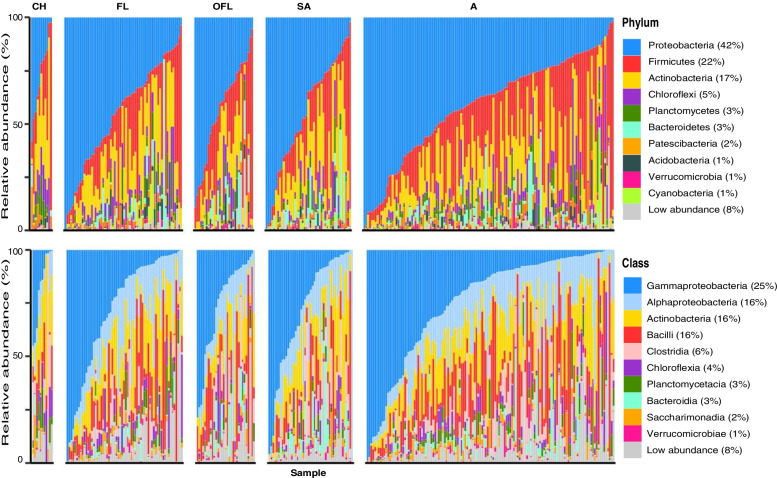
The relative abundance of bacterial **A** phyla and **B** classes in Seychelles warbler faecal samples. Each column represents a single faecal sample (281 faecal samples, collected from 224 Seychelles warblers). Samples are separated by age class: CH = chick, FL = fledgling, OFL = old fledgling, SA = sub-adult, A = adult. *Y*-axis shows the relative abundance (%) of the 10 most abundant bacterial taxa. All other taxa are collapsed into the low abundance category.

The overall bacterial phyla and class profile of the Seychelles warbler GM was similar to other passerine species [108]. We identified 40 bacterial phyla across the 281 samples combined; however, of these, *Proteobacteria* (42% of total reads), *Firmicutes* (22%), and *Actinobacteria* (17%) dominated, with all other phyla being present at lower relative abundances (summing to <5% of the total read count). The dominant bacterial classes were *Gammaproteobacteria* (25%), *Alphaproteobacteria* (16%), *Actinobacteria (*16%), *Bacilli* (16%), and *Clostridia* (6%) (Fig 1). At lower taxonomic levels, we identified 126 classes, 372 orders, 745 families, 1,827 genera, 2,586 species and 34,869 ASVs across the 281 samples combined.

The core microbiome was further characterised at the family level by extracting bacterial families that appeared in at least 50% of samples with a minimum relative abundance of 0.1% (Additional file 1: Table S4). This resulted in the detection of 28 core families, with ASVs from these families making up 75% of all reads. Of the core families, eight were present in at least 80% of samples, and four accounted for >5% of all reads; this latter group consisted of *Enterobacteriaceae* (23% of total reads), *Streptococcaceae* (10%), *Rhizobiaceae* (6%), and *Enterococcaceae* (5%). Of the assigned genera, 20 were present in at least 50% of samples and ASVs from these genera made up a total of 28% of all reads. However, of these, only two genera (*Microbacterium* and *Enterococcus*) were present in more than 80% of samples.

Despite the presence of a core microbiome, the abundance of individual bacterial taxa was highly variable across individuals as is typical in a wild species (Fig. 1) [109–111]. Additionally, there was considerable individual variation in alpha diversity when measured as Chao1 (mean = 323.2 ± 14.63 SEM), Shannon (mean = 4.0 ± 0.07), and Faith’s phylogenetic diversity (mean = 18.8 ± 0.62). Individual repeatability of the GM was tested using samples taken from the same individual during the same catch or field season, and three metrics of diversity (Shannon, unweighted, and weighted UniFrac). Pairwise beta diversity distances between different individuals were significantly greater (more dissimilar) than within-individual comparisons of samples taken during the same catch or field season (*n* = 51, unweighted UniFrac; χ_1_ = 21.28, *P* <0.001: weighted UniFrac; χ_1_ = 6.06, *P* = 0.014; Additional file 1: Fig S3). Likewise, pairwise Shannon diversity distances between different individuals were significantly greater (more dissimilar) than within-individual comparisons (*n* = 49, χ_1_ = 4.51, *P*= 0.034; Additional file 1: Fig S3). Note that in this analysis, two samples with unusually low Shannon diversity (0.9 compared to a mean of 4.0) were not included.

Repeatability of GM sequencing for the same sample was tested using three metrics of diversity (Shannon, unweighted, and weighted UniFrac). As expected, pairwise distances between samples were significantly greater (or more dissimilar) than within-sample comparisons, i.e., pairwise distances when gDNA from the same sample was sequenced twice (*n* = 37, *P* < 0.001) (Additional file 1: Fig S4).

### MHC characteristics

244 individuals were successfully genotyped at MHC-I exon 3 and MHC-II exon 2 genes. The repeatability of MHC-I genotypes was 95.0% and of MHC-II was 90.1%, based on 26 and 24 duplicate samples, respectively (Additional file 1: Table S2, S3). The great reed warbler positive control sample had four MHC-I and four MHC-II alleles—all of which mapped with 100% similarity to previously sequenced great reed warbler MHC alleles.

**Fig. 2:**
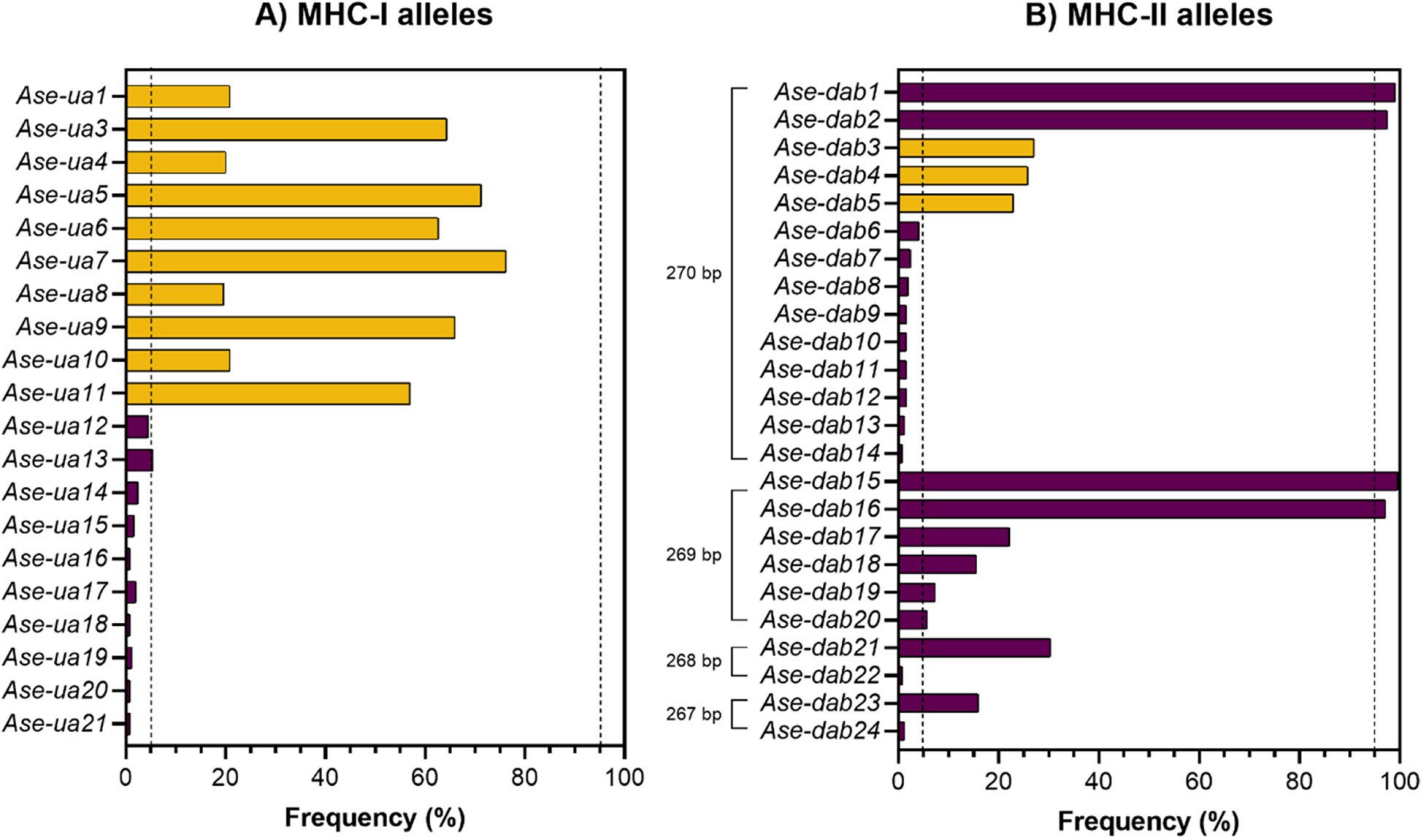
Variation in MHC-I exon 3 and MHC-II exon 2 in 244 Seychelles warblers. Each bar represents the frequency (%) of each **A** MHC-I allele and **B** MHC-II allele. Bars represent MHC alleles included (yellow) or not included (purple) in presence/absence analysis. Dashed lines indicate 5 or 95% frequency cut-offs

On average, individuals had 5.0 MHC-I alleles: 2–7 alleles per individual. Of these, 10 MHC-I alleles were present in >5% but <95% individuals, and another 10 were present in ≤5% of individuals (Fig. 2). Nine of the ten common alleles matched previously sequenced alleles [53], with an average of 98% sequence similarity. *Ase-ua2* was not present in the current sequencing cohort. When comparing 29 individuals also genotyped using reference strand-mediated conformation analysis [73], and excluding *Ase-ua2*, there was 95% similarity between genotyping methods.

Including all MHC-II alleles, individuals had on average 5.8 alleles (range 3–11) out of a total of 24 alleles (Fig. 2a). However, of these 24 MHC-II alleles, only 14 were of the full exon length (270 bp), six alleles had a 1-bp deletion (269 bp in length), two alleles had a 2-bp deletion (268 bp) and two alleles had a 3-bp deletion (267 bp). Of the 10 alleles which contained indels, three of these also contained stop codons, and all were missing the Cys74 residue, which along with Cys10 residue creates a disulphide bridge, which is important for conformation of the mature MHC protein; therefore, these alleles were removed as putative pseudo- or non-functional alleles. Concentrating on putatively functional MHC-II alleles, there were 2.9 alleles on average per individual (range 1–5 alleles per individual). Of these, only three were present in >5% but < 95% of individuals (Fig. 2b). Of the other putatively functional alleles, two were present in virtually all individuals, while nine alleles had a frequency <5%.

For downstream analysis, only alleles of the full, correct length (i.e. MHC-I: 262 bp, MHC-II: 270 bp) were included when calculating diversity or for presence/absence. Again, for the presence/absence of alleles, only alleles present in >5% but <95% of individuals were included. Of those alleles included in the final presence/absence analyses, all 10 of the MHC-I and three of the MHC-II alleles translated into unique amino acid sequences.

### The effect of MHC and other host variables on GM alpha diversity

**Fig. 3:**
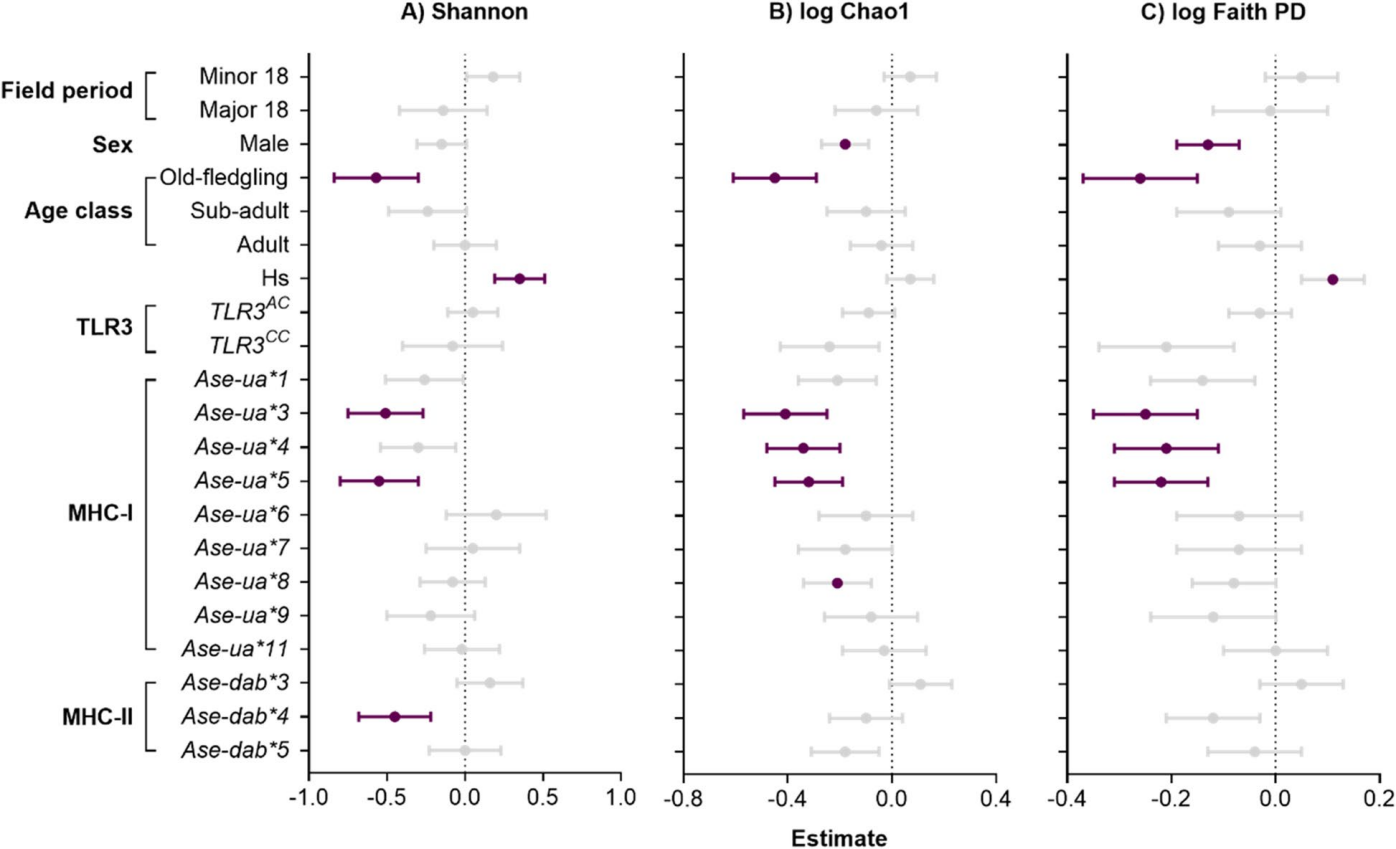
Effects MHC alleles, *TLR3* genotype, genome-wide heterozygosity, and host variables on alpha diversity in 195 Seychelles warblers. Alpha diversity metrics are **A** Shannon diversity, **B** Chao1, and **C** Faith’s phylogenetic diversity (PD). Estimates and standard errors are based on linear conditional model-averaged estimates. An estimate >0 indicates increased alpha diversity, while <0 indicates decreased alpha diversity. Significant terms (*P* < 0.05) are highlighted in purple, and terms approaching significance (*P* < 0.1) are indicated with a purple point. Estimates are in reference to MHC allele = absent, *TLR3* genotype = *TLR3* ^AA^, sex = female, age class = fledgling, field period = major 2017

The presence of four MHC alleles was associated with reduced diversity and richness of the GM (Fig. 3). Individuals with the *Ase-ua5* allele had significantly lower alpha diversity for all calculated metrics, compared to individuals without (Additional file 1: Table S6, Fig 3), indicating that *Ase-ua5* negatively influences the richness (*β* ± SE = −0.32 ± 0.13, *z* = 2.40, *P* = 0.016), evenness (*β* ± SE = −0.55 ± 0.25, *z* = 2.22, *P* = 0.027) and the phylogenetic diversity (*β* ± SE = −0.22 ± 0.09, *z* = 2.36, *P* = 0.018) of the GM. Likewise, the presence of the *Ase-ua3* allele was also associated with a decrease in Shannon diversity (*β* ± SE = -0.51 ± 0.24, z = 2.16, *P* = 0.031), Chao1 richness (*β* ± SE = −0.41 ± 0.16, *z* = 2.62, *P* = 0.009: Additional file 1: Table S6a, Fig 3) and phylogenetic diversity of the GM (*β* ± SE = −0.25 ± 0.10, *z* = 2.39, *P* = 0.017: Additional file 1: Table S6a, Fig. 3). The presence of the *Ase-ua4* allele was associated with reduced GM richness (*β* ± SE = −0.34 ± 0.14, *z* = 2.35, *P* = 0.019) and phylogenetic diversity (*β* ± SE = −0.21 ± 0.10, *z* = 2.15, *P* = 0.032; Additional file 1: Table S6a, Fig. 3), but there was no significant difference in the evenness of the GM between individuals with or without the *Ase-ua4* allele (Additional file 1: Table S6a, Fig 3). None of the remaining MHC-I alleles or *TLR3* genotype were associated with alpha diversity (Additional file 1: Table S6a, Fig. 3). Likewise, most MHC-II alleles were not associated with changes in GM diversity. However, the presence of one allele, *Ase-dab4*, was associated with a reduction in Shannon diversity (*β* ± SE = −0.45 ± 0.23, *z* = 1.98, *P* = 0.048; Additional file 1 Table S6a, Fig 3), but not Chao1 richness or Faith’s phylogenetic diversity (Additional file 1: Table S6a, Fig 3). There was no significant effect of MHC-I or MHC-II diversity, or diversity^2^ on alpha diversity (Additional file 1: Table S6b). By contrast, individual heterozygosity was positively associated with Shannon diversity (*β* ± SE = 0.35 ± 0.16, *z* = 2.21, *P* = 0.027; Additional file 1: Table S6a, Fig. 3), but not Chao1 or Faith’s phylogenetic diversity, though these both showed the same pattern.

Males had reduced phylogenetic diversity compared to females (*β* ± SE = −0.13 ± 0.06, *z* = 2.13, *P* = 0.034, Additional file 1: Table S6, Fig 3), and the same pattern was evident when comparing richness. There was also an association between GM diversity and age, with old fledglings having reduced evenness, richness and phylogenetic diversity compared to adults (Shannon; *β* ± SE = −0.57 ± 0.27, *z* = 2.10, *P* = 0.036: logChao1: *β* ± SE = -0.45 ± 0.16, z = 2.62, *P* = 0.009; logFaiths: *β* ± SE = −0.26 ± 0.11, *z* = 2.45, *P* = 0.015; Additional file 1: Table S6, Fig 3). There was no association between GM diversity and field period (Additional file 1: Table S6a, Fig. 3), suggesting that environmental variation across field periods had little influence on the observed variation in alpha diversity values across individuals.

### The effect of host variables on GM composition

**Fig. 4:**
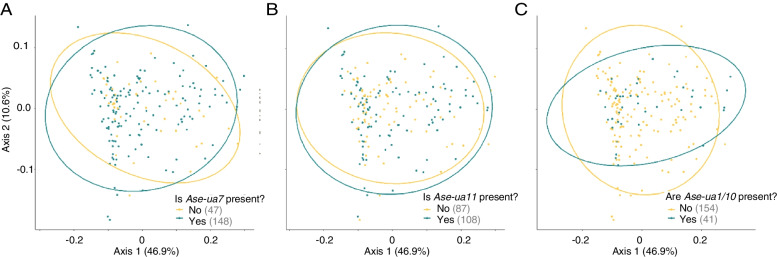
Beta diversity of Seychelles warbler gut microbiome composition according to the presence of three MHC-I alleles. The principal coordinate plots are based on weighted UniFrac distances according to the presence or absence of the MHC-I **A** *Ase-ua7* allele, **B** *Ase-ua11* allele, or **C** *Ase-ua1/10* allele. Points represent a single faecal sample from a single individual (*n* = 195). Sample sizes are specified in brackets in the legend, and colours indicate the presence (blue) or absence (yellow) of the MHC-I allele. Ellipses represent a 95% confidence interval around the cluster centroids

In addition to effects on alpha diversity, compositional differences in the GM of individuals with, or without, specific MHC-I alleles were identified, although the alleles that showed an effect were not the same as those associated with shifts in GM alpha diversity. PERMANOVA tests showed that the overall composition of the GM was significantly different for individuals with the *Ase-ua7* allele, the *Ase-ua11* allele, or for *Ase-ua1* (and *Ase-ua10* as these alleles were co-occurring) versus those without them (Table 1, Fig. 4), but only when weighted UniFrac distances were used as a beta diversity metric. None of the differences detected in PERMANOVA tests were due to differential dispersion (all betadisper tests: *P* > 0.05), indicating that the results reflected differences in mean values across groups. However, although statistically significant, the presence/absence of specific alleles only explained a minimum of 1.4% and a maximum of 1.7% (per allele) of the variation in GM composition, suggesting that each allele only had a small influence on overall GM composition (Table 1). The remaining MHC-I and MHC-II alleles, as well as MHC-I and MHC-II diversity (or diversity^2^) had no effect on GM composition (Table 1). Additionally, *TLR3* genotype and *H*_s_ were not associated with any of the beta diversity metrics (Table 1).

Age class was associated with a compositional shift in the GM in PERMANOVAs based on unweighted UniFrac distances (Table 1) and explained 1.9% of the variance in GM composition. Based on the Principal Coordinate analysis plots, this difference was due to old fledglings being slightly more differentiated compared to other age classes (Additional file 1: Fig S5). However, this effect was absent in models based on weighted UniFrac, which takes the abundance of ASVs into account (Table 1). This suggests that the changes in composition with age class may be due to differences in the presence/absence of different bacterial taxa in the GM, rather than differing abundances of the same taxa. There were no differences in beta diversity between males and females (Table 1). There were significant compositional differences in the GM between field periods, which overall explained either 1.7 or 2.1% variance for unweighted and weighted UniFrac distance, respectively (Table 1).

### The influence of host variables on the abundance of specific ASVs

**Fig. 5:**
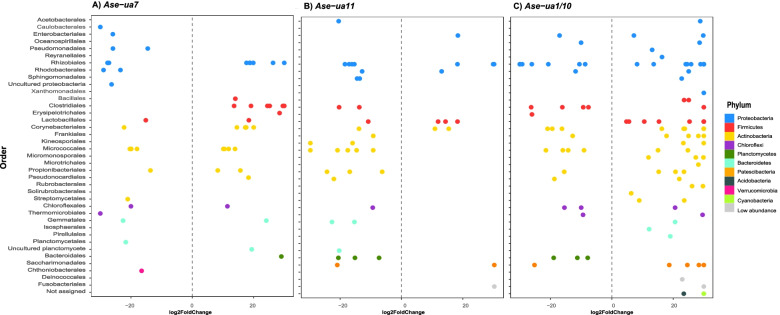
Differentially abundant ASVs in the gut microbiomes of Seychelles warblers, according to the presence/absence of the MHC-I alleles **A** *Ase-ua7*, **B** *Ase-ua11,* or **C** *Ase-ua1/10*. ASVs are grouped at the level of bacterial order and coloured according to bacterial phylum. Differential ASV abundance was assessed using negative binomial Wald tests, and *P* values were adjusted using the Benjamin and Hochberg false-discovery rate correction with a significance cut-off of *P* < 0.01. ASVs shown with a log_2_-fold change greater than zero are significantly more abundant in individuals without this allele and ASVs with a log_2_ fold change smaller than zero are significantly more abundant in individuals with a copy of this allele.

The co-occurring *Ase-ua1/10* alleles were associated with the greatest change in ASV abundance, with 67 ASVs (across 31 bacterial orders) being significantly more abundant when these alleles were absent and 32 ASVs (across 15 orders) being more abundant when they were present (Additional file 3; Fig. 5c). Fewer taxa were differentially abundant between groups of individuals with/without *Ase-ua11*. In this instance, 12 ASVs (across 7 orders) were significantly more abundant when the allele was absent, and 32 ASVs (across 17 orders) were more abundant when the allele was present (Additional file 3; Fig. 5b). Overall, 30 ASVs (across 13 orders) were significantly more abundant when the allele *Ase-ua7* was absent and 22 ASVs (across 16 orders) were more abundant when the allele was present (Additional file 3; Fig. 5a).

Many ASVs were significantly less abundant in old fledglings compared to other age groups (old fledglings compared to fledglings: 24 vs 150; old-fledgling compared to sub-adults: 59 vs 134; old-fledglings compared to adults: 18 vs 168; Additional file 1: Fig S6). In comparison, the numbers of differentially abundant taxa between other age groups were more even (fledglings compared to sub-adults: 47 vs 55; fledglings compared to adults: 22 vs 33; sub-adults compared to adults: 22 vs 86; Additional file 1: Fig S6).

Concentrating on extrinsic associations with GM, 225, 227, and 144 ASVs were significantly differentially abundant between the three field periods, respectively (Additional file 1: Fig S7). The majority of ASVs were underrepresented in the minor 2018 season compared to either major season (60 in the minor 2018 vs 167 in the major 2017 season, and 37 in the minor 2018 season vs 188 in the major 2018 season). Of these, 150 ASVs from the minor season differed in abundance across analyses.

## Discussion

In this study, we screened GM variation and MHC class I and II characteristics of individuals in a natural population of the Seychelles warbler. This enabled us to assess how the diversity and composition of the bacterial component of the GM is associated with individual immunogenetic variation i.e. MHC and *TLR3* genotypes. Our results indicate that differences in GM diversity are associated with the presence of certain MHC alleles (specifically, lower diversity is associated with four of the 13 tested MHC alleles). Furthermore, differences in GM composition are associated with the presence/absence of four (different) MHC-I alleles, including the differential abundance of certain bacterial taxa. While we found no effect of MHC diversity or *TLR3* genotype on the GM, we did find a positive association between bacterial GM diversity and individual genome-wide heterozygosity. Lastly, GM characteristics were also associated with several other host specific or extrinsic variables, namely sex, age, and sampling period.

### MHC variation in the Seychelles warbler

Here, we screened variation at the MHC-I exon 3 and MHC-II exon 2 genes using next-generation sequencing. We found reduced functional allelic diversity at MHC-II compared to MHC-I, consistent with what has been found in other passerines [112]. Previous studies on the Seychelles warbler have provided evidence that balancing selection has maintained variation at both the MHC-I [53] and MHC-II [54]. However, the latter study did not fully resolve individual MHC-II characteristics because of difficulties with the cloning and reference strand-mediated conformation analysis techniques used. In the present study, we were able to confirm (class-I) and fully characterise (class-II) individual MHC variation. Our results, showing that variation has been maintained at both sites in this species despite reduced genome-wide variation [51], support the idea that balancing selection is maintaining variation at both MHC classes. Given the MHC’s role in antigen detection, this is likely to be pathogen-mediated selection.

### GM variation is associated with MHC variation

We found that GM diversity was negatively associated with the presence of three (of 10) MHC-I alleles (*Ase-ua3*, *Ase-ua4*,* Ase-ua5*) and one (of three) MHC-II alleles (*Ase-dab4*). All three MHC-I alleles were consistently associated with a reduction in GM bacterial richness and phylogenetic diversity. *Ase-ua3* and *Ase-ua5* were additionally associated with reduced evenness in the GM. This suggests that these alleles may lead to the selective elimination of certain bacterial taxa from the gut, resulting in a reduced community of species with a narrower phylogenetic range. This is similar to another study that identified MHC-II motifs associated with reduced GM diversity in threespine sticklebacks [43]. It is likely that MHC-II alleles—such as *Ase-dab4* in the Seychelles warbler—directly impact GM diversity, since MHC-II molecules are produced in antigen-presenting cells, which are abundant in the lamina propria behind the gut epithelium [22]. Such dendritic cells can extend between epithelial cells where they phagocytose particles, including microbes, from the gut lumen [113]. Antigens from these microbes are then exported to the cell surface by MHC-II molecules, so that they can be presented to B and T cell populations [114] and, if recognised, instigate an immune response.

Our study expands on previous work by investigating how variation at MHC-I genes impact GM variation in a natural population (see also [42]). Laboratory studies comparing transgenic and wild-type rats [39] have previously shown that the expression of human MHC-I genes (specifically HLA-B27, typically associated with arthritis) alters GM composition and that this may be related to immune-mediated disease state. Likewise, in congenic mice [60], the lack of MHC-I-mediated antigen presentation resulted in shifts in composition and structure of the GM. In addition to the simplification of the GM in captive populations [6], animal models are typically engineered to be homozygous/heterozygous or to have different expression at specific alleles. In comparison, natural populations normally contain a diverse array of alleles and allelic combinations, and are exposed to a wider range of microbial taxa; thus, biologically relevant indirect effects of host genes on the GM may not be observed in captivity. This is particularly important when considering associations between GM characteristics and individual MHC-I variation. MHC-I molecules typically respond to intracellular microbes, rather than extracellular microbes, and play a central role in anti-viral and anti-tumour immunity [31]. As such, we would not necessarily expect these molecules to recognise bacterial antigens, although cross reactivity via the presentation of exogenously derived antigens can occur [115]. Instead, MHC-I variation may indirectly affect GM characteristics by impacting other aspects of the host’s health and physiology. In the Seychelles warbler, we know that survival is associated with a specific MHC-I allele (*Ase-ua4*) [58] and, although we do not know what drives this effect (i.e. we have not identified the selective factor responsible), any such interaction could also shape changes in the GM. In our study, the *Ase-ua4* allele was associated with a reduction in GM richness and phylogenetic diversity but did not significantly change the composition of the GM. Numerous studies have linked MHC-I variation with susceptibility to malarial infection in passerines [116, 117] and other taxa [32]. Malaria infections alter the GM via disruption to immune homeostasis [118] and could play a role in the Seychelles warbler, in which a single strain of the malarial parasite (*Haemoproteus nucleocondensus)* has been identified [119]. Alternatively, MHC-I might alter an individual’s susceptibility to another, as yet, unidentified pathogen, that could also drive differences in the diversity of the GM [47, 120].

As well as MHC alleles directly, or indirectly (through fitness) driving the differences in the GM we see here, it is possible that these associations arise due to other causes. GM taxa have been shown to be heritable [121], and other genes have been associated with GM variation in wild populations [19]. It may also be that variation at other loci in linkage disequilibrium with the MHC genes investigated here could drive the associations observed here. This is especially important to consider in bottlenecked species, such as the Seychelles warbler, where there will be considerable linkage disequilibrium throughout the genome. However, given the important role of the MHC genes in the acquired immune response, and because the alleles identified encode putatively functional variation in the peptide binding region of the MHC molecules, it is logical to suspect that the MHC variation is involved in driving the changes in GM variation seen here.

Given the negative, but lack of positive, associations identified between MHC alleles and GM alpha diversity, it is surprising that there was no significant effect of overall MHC diversity on the GM. One might expect the cumulative negative effect of each allele (Fig. 3) to cause at least a weak negative correlation between GM diversity and MHC diversity. However, with the multitude of factors involved in determining both the host’s GM and immune response, this lack of association could simply be due to limited power to detect weak effects, as is often the case when examining associations between immunocompetence and MHC variation [122]. Assessing how the functional divergence of MHC alleles within an individual—which provides information about the range of antigens that can be detected [123]—rather than just the number of MHC genes has provided additional resolution in other MHC studies e.g. [124]. This approach can provide extra clarity in species with high MHC diversity where multiple alleles may be functionally similar with overlapping antigen binding properties [125, 126] particularly when considering the diversity of bacterial taxa that are present within the GM. However, in the bottlenecked population of the Seychelles warbler, the very limited number of alleles present in the MHC-I are all highly divergent and cannot be further reduced into functional supertypes. This is likely because functionally divergent MHC alleles were selected for through the bottleneck this species underwent [53]

We also observed compositional differences in the GM associated with four MHC-I alleles (*Ase-ua7*, *Ase-ua11,* and the co-occurring *Ase-ua1/10* alleles) but not with any MHC-II alleles. Alleles linked to compositional differences were different to those that were negatively associated with GM alpha diversity. This pattern could arise if different ASVs are tolerated, or removed by MHC-mediated responses, but overall GM richness remains similar across individuals with or without a particular allele, causing compositional but not alpha diversity differences. In contrast, where a greater number of taxonomically similar ASVs and/or less abundant taxa—which may have a reduced impact on compositional differences—are removed by MHC-mediated responses, this may result in reduced alpha diversity but a largely similar composition in individuals with, compared to those without, these alleles. The biggest compositional shift was associated with *Ase-ua1/10*, whose presence/absence caused the greatest number of differentially abundant ASVs. This is perhaps not surprising, as individuals with both alleles would be able to recognise a larger number of antigens, thus providing a broader immune response compared to a single allele. However, the presence/absence of *Ase-ua1/10* only explained 0.5–1.4% (depending on the metric of beta diversity) of the variance in GM composition suggesting that, separately, each allele has a relatively small impact on the GM overall.

Several ASVs were not assigned beyond the level of bacterial family and many bacterial taxa have not been fully characterised, making it difficult to draw conclusions about the functional significance of compositional changes in the GM for the host. However, there were several potentially interesting, shared candidate taxa that were differentially abundant between individuals with/without the *Ase-ua1/10* and *Ase-ua11* alleles. For example, individuals with these alleles had a reduced abundance of ASVs from the order *Lactobacillales*, a lactic acid-producing bacterial taxon, generally thought to be a beneficial member of the GM. Indeed, members of this order are used as probiotics in poultry farms to boost the immune response of chickens [127]. In contrast, individuals with the *Ase-ua1/10* and *Ase-ua11* alleles had an increased abundance of ASVs from *Bacteroidales*, an order commonly associated with chronic intestinal inflammation [128]. Two of these ASVs were from the genus *Bacteroides*, while species from this genus can be mutualistic, opportunistic pathogenic infections can occur in humans and other animals [129]. Likewise, a third ASV from the genus *Alistipes* has a pathogenic role in various human and animal diseases [130]. The patterns of change associated with *Ase-ua7* were different to those arising from the presence/absence of *Ase-ua1/10,* and *Ase-ua11*, with fewer ASVs from the orders *Lactobacillales* and *Bacteriodales* being differentially abundant. Instead, several ASVs in the order *Clostridiales* were significantly more abundant when the *Ase-ua11* or *Ase-ua1/10* alleles were present (and less abundant when the *Ase-ua7* allele was present), suggesting that this order could have been selectively tolerated or that ASVs in this order proliferated when other competing taxa were removed.

Direct tolerance of gut commensals by the host immune system can occur by several means, secretions produced by the microbes themselves can induce tolerance by the host immune system, or tolerogenic responses can be genetically encoded by the host [131]. For example, genetically encoded tolerance mechanisms can occur through differential MHC expression on key tolerogenic inducing cells, such as the group 3 innate lymphoid cells [132]. Immunoglobulin A repertoire in the gut can be controlled by MHC genotype, mediating the response against commensal microbes [60]. Mucosal dendritic cells can also induce tolerogenic T and B cell responses, and regulatory T cells can directly recognise, and tolerate, commensal antigens [61, 62]. However, it is not clear how the presence of different polymorphisms in the MHC binding region could facilitate these mechanisms. More likely, coevolution between host and GM resulted in the MHC repertoire that has evolved to tolerate commensal taxa, with MHC alleles that eliminate commensal antigens being under weakened (or even negative) selection compared to those that eliminate pathogenic microbial antigens.

Cumulatively, the variance in composition explained by overall MHC allele presence or absence was low (6.3% or 8.9% for unweighted or weighted UniFrac, respectively). However, this is not unusual when investigating the factors that influence GM composition across individuals within a single population; for example, environmental and host factors explained between 0.4 and 10% variance in red squirrels (*Tamiasciurus hudsonicus*) [110]. Even sampling period, the most significant predictor of beta diversity in our study, explained only 2% of variation in the GM. One explanation for the low level of explained variance could be the greater presence of transitionary microbiota in the avian gut i.e. dietary, or environmental microbes that are ingested and pass through the intestine without interacting with the host [133]. Adaptations for flight have placed constraints on avian morphology, leading to shorter intestinal lengths, and consequently, shorter food retention times [134]; this may reduce the potential for bacterial species to adapt to the avian gut and to variation in host ecology. Secondly, if many bacterial taxa carry out the same function in the host gut, there could be a high turnover of species without any consequences for the host [135]. This can give rise to high inter-individual variation in the GM and may explain why the variables analysed here (or indeed in many within-population studies of the GM) explain a low proportion of the overall variance. Indeed, functional GM diversity may be more important than species diversity for host fitness [136]. To address this, future work incorporating metagenomic analysis would allow greater resolution of bacterial species and a more accurate assessment of the functional composition of the GM [137].

Apart from the acquired immune response, the GM may also be affected by the innate immune response (underpinned by genes such as TLRs) [27]. However, we detected no effect of *TLR3* genotype (one of the few TLRs to have functional variation in this system [55]) on the GM; this is despite survival and reproductive success being significantly associated with *TLR3* variation in the Seychelles warbler [50]. This is perhaps not surprising, given *TLR3*’s role in recognising viral dsRNA [138], rather than any bacterial conserved structures.

Individual heterozygosity was positively correlated with GM bacterial richness in the Seychelles warbler, though this was not associated with differences in GM phylogenetic diversity or composition. A decrease in individual heterozygosity (or increase in homozygosity) may reflect increased inbreeding. In the Seychelles warbler, increased inbreeding is associated with poorer individual condition (via reduced telomere length [56]) and reproductive success, with maternal homozygosity negatively predicting offspring survival in years with high mortality [57, 139]. Thus, we may be detecting an indirect effect of increased inbreeding, whereby the decreased fitness or health of individuals in turn negatively impacts GM diversity [140]. However, it is also possible that this association could be driven by reduced heterozygosity of currently unknown, specific functional loci directly reducing GM diversity. In future studies, it could be informative to use either quantitative trait locus mapping [141], or genome-wide association studies to identify candidate genes associated with GM variation, e.g. [19, 142]. The Seychelles warbler could be particularly useful for this, as it underwent a bottleneck in the 1960s, resulting in a 25% reduction in genome-wide variation [51], thus making it a more tractable study system in which to disentangle the associations between host genetic variation and the GM.

It is difficult to assess the impact of the identified relationships between GM variation and MHC alleles on host fitness. Typically, greater GM alpha diversity is thought to be beneficial, as it correlates with increased health and survival in humans [24]. However, other studies have shown that high alpha diversity can indicate dis-regulation and GM instability [143]. Similarly, the function of many bacteria in the GM of wild animals is unknown. Given the key role that MHC genes play in pathogen resistance, it is possible that the observed negative correlation between GM alpha diversity and presence of specific MHC alleles is beneficial to the host. For example, Seychelles warblers with the *Ase-ua4* allele had reduced GM alpha diversity, and this same allele conferred a survival advantage in individuals [50,58]. To fully unpick the consequences of these GM/MHC relationships in the Seychelles warbler, longitudinal data and analyses accounting for within- and between-individual differences are needed to fully test whether there are fitness differences between individuals with different MHC alleles and GM characteristics. This is no small undertaking, and will require extensive and powerful datasets, which are not yet available.

### The GM may drive the evolution of immune genes

Variation in the GM can affect traits important to the host’s own fitness [144], including host immune function [145], the severity of diseases [146] and, ultimately, survival [109, 147], and this could provide the potential for evolutionary adaptation in the host [148]. Balancing selection is thought to be central in maintaining diversity at MHC genes [149–151]. Thus, the GM could act as a selective pressure, shaping host phenotypes (reviewed in [152, 153]), ultimately resulting in host-microbiome co-evolution and adaptation, or speciation [9, 154]. For example, if components of the GM interact with MHC variation, leading to the differential selection of MHC alleles, this could explain how variation at MHC genes has been maintained in the previously bottlenecked Seychelles warbler [51, 53], despite the very limited macroparasite fauna in this population [54]. We detected no effect of MHC diversity (or optimality) on the GM, and therefore no evidence of MHC heterozygote advantage in relation to the GM [155]. However, specific alleles were associated with differences in GM composition; this is consistent with either rare allele [156] or fluctuating selection [32] mechanisms, although differentiating between the two is extremely difficult [35]. Identifying the function of GM taxa that are associated with MHC alleles, whether they be pathogenic, beneficial, or commensal, could help infer the significance and direction of these associations.

### Effects of age, sex and field period on the GM

In addition to genetic factors, several other key variables influenced the GM. Our results indicated a relationship between GM composition and age class in the Seychelles warbler. Old fledglings had reduced GM alpha diversity and compositional differences compared to all other age group comparisons (which did not differ from one another). In the Seychelles warbler, old fledglings are newly independent and start to forage for themselves and so may be eating different—perhaps lower quality—food items compared to older birds. This may explain the reduced number of differentially abundant taxa present in this age group, compared to others, including a reduced abundance of ASVs in the order *Planctomycetes*, which are typically transient colonisers of the gut (but see [157]). Alternatively, exposure to stress via glucocorticoids alters host GM in other species [16]. Thus, increased stress in young individuals as they encounter new situations and pathogens could contribute to differences between age groups. Indeed, mortality is greatest during the first year of life in the Seychelles warbler [57].

While sex is an important determinant of individual variation in natural populations, its importance as a driver of GM variation varies across vertebrate species [10, 14, 110, 158]. Sex was only associated with a minor difference in the GM of the Seychelles warbler, with males having marginally reduced diversity, but no difference in composition compared to females. It is, perhaps, not surprising that the effect of sex on the GM was so limited, given that Seychelles warblers of both sexes have the same diet and exhibit limited differences in morphology and behaviour. In threespine sticklebacks, GM–MHC associations were sex-dependent [43]; however, we found no evidence of this in the Seychelles warbler.

Within a species, seasonal changes in diet can be an important factor driving GM variation [12, 110, 159]. In the Seychelles warbler, field period explains 1.7–2.1% of the variance in GM composition. Although the temperature on Cousin Island is relatively stable, there are measurable differences between seasons and years [160], which could lead to variation in the type and abundance of insect prey species. This may explain the observed difference in GM composition, but not diversity, between field periods. For example, mean island-wide territory quality increased by 80% in the major 2017 field period and 75% in the minor 2018 field period, compared to the later major 2018 field period. Alternatively, increases in food availability between seasons could also act indirectly on the GM by buffering individuals against stress or susceptibility to pathogens.

## Conclusions

Our results show that variation has been maintained at MHC-I and MHC-II genes in the Seychelles warbler, and that the presence of specific alleles, but not MHC diversity, was associated with differences in GM diversity and composition. It is possible that such GM–MHC interactions might explain previous results in this population showing that specific MHC alleles are associated with higher survival. However, further longitudinal data are needed to establish whether these associations equate to fitness differences between individuals and to better understand host immunogenetic–GM coevolution.

## Supplementary Information

**Additional file 1: Table S1.** Primer sequences used for MHC sequencing. Degenerate bases are shown according to IUPAC codes: Y = C/T, N = any base. **Table S2.** Repeatability of MHC-I (*n* = 26) and MHC-II (*n* = 24) genotyping for different dominant frequency thresholds. Minimum amplicon frequency was kept constant at 0.3%. Threshold with the greatest repeatability is in bold. **Table S3.** Repeatability of MHC-I (*n* = 26) and MHC-II (*n* = 24) genotyping for different minimum amplicon frequencies. Minimum dominant frequency threshold was kept constant at 25%. Minimum amplicon frequency with the greatest repeatability for each MHC class is in bold. **Table S5.** Core families present in 281 faecal samples, collected from 224 Seychelles warblers. Core microbiome is defined as bacterial families that appeared in at least 50% of samples, with a minimum relative abundance of 0.1%. Total number of reads, and % of all reads are included. **Table S5.** The effect of host-associated variables on gut microbiome diversity in the Seychelles warbler (n = 195). GLMMs for three metrics of alpha diversity: Shannon diversity, Chao 1(log transformed) and Faiths phylogenetic diversity (log transformed): A) including the presence/absence of MHC alleles or, B) MHC diversity. A Linear model was used to generate conditional model-averaged estimates (β), their standard error (SE), z value, *P* value, and relative importance (ω) are shown for all predictors featuring in the top model set (ΔAIC_c_ ≤ 7). All continuous factors were standardised. Estimates are in reference to MHC allele = absent, *TLR3* genotype = *TLR3*^AA^, sex = female, age class = fledgling, field period = Major 2017. Significant terms are in bold and underlined. *** *P* < 0.001, ** *P* < 0.01, * *P* < 0.05. **Figure S1.** (A) Sample completeness and (B) rarefaction curves in Seychelles warbler faecal samples. Each line represents a single faecal sample (281 faecal samples, collected from 224 Seychelles warblers). Curves were generated using the R package iNEXT 2.0.20, with 50 bootstrap replicates per sample. The dashed line represents the number of reads used as a cut-off for retaining samples in downstream analysis (all samples with fewer than 10,000 reads were removed). **Figure S2.** Prevalence and total abundance of all ASV’s separated by phylum. Each phylum is shown in a separate plot, and a different colour. Dashed lines represent the values used as cut-offs for filtering rare taxa before alpha and beta diversity analyses (minimum abundance = 50), and additional filtering for beta diversity (prevalence threshold = 2.5%). **Figure S3.** Individual repeatability of alpha and beta diversity measures in the Seychelles warbler. This was tested by sequecnoing multiple samples taken from the same individuals; these samples were collected during the same field season (n = 115 faecal samples from 51 individuals. Pairwise Euclidean distances were calculated between samples taken from different individuals, versus those from within the same individual, in the same season for A). Shannon dissimilarity B) unweighted UniFrac dissimilarity and C) weighted UniFrac dissimilarity. Boxes span the interquartile (25% - 75%) range. Whiskers extend to 1.5 times the interquartile range. The median is marked by a horizontal line and the mean is marked by a diamond. Dark blue points in A) indicate pairwise comparisons involving two outliers. Significant differences are shown, and *P*-values are derived from Kruskal–Wallis tests: *** *P* < 0.001, * *P* < 0.05. **Figure S4.** The repeatability of sequencing methods. This was tested by sequencing 37 faecal samples taken from individual Seychelles warblers twice. A) Relative abundance (%) of the 10 most abundant taxa at the phylum level for the 37 duplicated samples. Each column represents one sample, black lines separate duplicated samples. All other taxa within each sample are collapsed into the low abundance category. B) The pairwise Euclidean dissimilarity between different samples, versus within pairs of duplicated samples (same DNA sequenced twice) for i. Shannon dissimilarity ii. unweighted UniFrac dissimilarity and iii. weighted UniFrac dissimilarity. Boxes span the interquartile (25% - 75%) range. Whiskers extend to 1.5 times the interquartile range. The median is marked by a horizontal line and the mean is marked by a diamond. Significant differences are shown, and P-values were derived from Kruskal–Wallis tests: *** *P* < 0.001. **Figure S5.** Beta diversity of Seychelles warbler gut microbiome composition in different age classes. The principal coordinate plots are based on A) unweighted UniFrac distances, and B) weighted UniFrac distances. Points represent a single faecal sample from a different individual (*n* = 195). Sample sizes are specified in brackets in the legend, and colours indicate the age class which was either fledgling (yellow), old-fledgling (green), sub-adult (indigo) and adult (purple). Ellipses represent a 95% confidence interval around the cluster centroids. **Figure S6.** Differentially abundant ASV’s in the gut microbiome of Seychelles warblers between different age categories (FL = fledgling, OFL = old fledgling, SA = sub-adult, A = adult). ASVs are grouped at the level of bacterial order and coloured according to bacterial phylum. Differential ASV abundance was assessed using negative binomial Wald tests and *P* values were adjusted using the Benjamini and Hochberg false-discovery rate correction with a significance cut-off of *P* < 0.01. ASVs shown with a log_2_ fold change greater than zero are significantly more abundant in the age classes on the left and ASVs with a log_2_ fold change smaller than zero are significantly more abundant in age classes on the right. **Figure S7.** Differentially abundant ASV’s in the gut microbiome of Seychelles warblers, between seasons. Comparisons are A) Major 2017 vs Minor 2018, B) Major 2018 vs Minor 2017, or C) Major 2017 vs Major 2018. ASVs are grouped at the level of bacterial order and coloured according to bacterial phylum. Differential ASV abundance was assessed using negative binomial Wald tests and *P* values were adjusted using the Benjamini and Hochberg false-discovery rate correction with a significance cut-off of *P* < 0.01. ASVs shown with a log_2_ fold change greater than zero are significantly more abundant in seasons on the left and ASVs with a log_2_ fold change smaller than zero are significantly more abundant in seasons on the right.

**Additional file 2.** The identity of 19 amplicon sequencing variants (ASVs) identified in the negative extraction controls. A taxonomic breakdown and the number of reads associated with each ASV is provided. ASVs were either filtered from the dataset before further analysis or retained. In the second tab is a detailed breakdown of number of reads with each ASV present in each sample, along with whether it is a negative control or faecal sample, and whether it was sequenced in the 1st, 2nd, or 3rd sequencing run.

**Additional file 3.** Differentially abundant ASVs (Padj < 0.01) in the gut microbiomes of Seychelles warblers, according to the presence/absence of the MHC-I alleles A) *Ase-ua7* B) *Ase-ua11* or C) *Ase-ua1/10*. Differential ASV abundance was assessed using negative binomial Wald tests and P values were adjusted using the Benjamini and Hochberg false-discovery rate correction with a significance cut-off of P < 0.01. ASVs shown with a log2-fold change greater than zero are significantly more abundant in individuals without this allele and ASVs with a log2 fold change smaller than zero are significantly more abundant in individuals with a copy of this allele.

## Data Availability

All 16S rRNA gene amplicon sequences have been submitted to the European Nucleotide Archive (ENA) database under the study accession number PRJEB45408. The 44 MHC alleles have been deposited at GenBank, accession numbers for MHC class I alleles are MZ509455-74, and for MHC class II alleles are MZ509475-98. All metadata, along with R scripts used to run analyses, are available in the Github Repository, https://github.com/Seychelle-Warbler-Project/Davies_2021_Microbiome.
