# Supplementary material for: Immunogenetic variation shapes the gut microbiome in a natural vertebrate population
Source: Microbiome. 2022 Mar 8;10:41. doi: 10.1186/s40168-022-01233-y (PMC8903650; doi:10.1186/s40168-022-01233-y)
Supplement: Supplementary file 2 — Additional file 1: Table S1. Primer sequences used for MHC sequencing. Degenerate bases are shown according to IUPAC codes: Y = C/T, N = any base. Table S2. Repeatability of MHC-I (n = 26) and MHC-II (n = 24) genotyping for different dominant frequency thresholds. Minimum amplicon frequency was kept constant at 0.3%. Threshold with the greatest repeatability is in bold. Table S3. Repeatability of MHC-I (n = 26) and MHC-II (n = 24) genotyping for different minimum amplicon frequencies. Minimum dominant frequency threshold was kept constant at 25%. Minimum amplicon frequency with the greatest repeatability for each MHC class is in bold. Table S5. Core families present in 281 faecal samples, collected from 224 Seychelles warblers. Core microbiome is defined as bacterial families that appeared in at least 50% of samples, with a minimum relative abundance of 0.1%. Total number of reads, and % of all reads are included. Table S5. The effect of host-associated variables on gut microbiome diversity in the Seychelles warbler (n = 195). GLMMs for three metrics of alpha diversity: Shannon diversity, Chao 1(log transformed) and Faiths phylogenetic diversity (log transformed): A) including the presence/absence of MHC alleles or, B) MHC diversity. A Linear model was used to generate conditional model-averaged estimates (β), their standard error (SE), z value, P value, and relative importance (ω) are shown for all predictors featuring in the top model set (ΔAICc ≤ 7). All continuous factors were standardised. Estimates are in reference to MHC allele = absent, TLR3 genotype = TLR3AA, sex = female, age class = fledgling, field period = Major 2017. Significant terms are in bold and underlined. *** P < 0.001, ** P < 0.01, * P < 0.05. Figure S1. (A) Sample completeness and (B) rarefaction curves in Seychelles warbler faecal samples. Each line represents a single faecal sample (281 faecal samples, collected from 224 Seychelles warblers). Curves were generated using the R package iNEXT 2 [file 40168_2022_1233_MOESM2_ESM.docx]

**Additional file 1:**

**Methods:**

*MHC sequencing*

All gDNA was normalised to 10 ng/ul. PCR1 amplification occurred in a total volume of 20 µl and used 2 µl gDNA (20 ng), 2 µl of forward and reverse MHC specific primers (5 µM, Additional file 1: Table S1), 10 µl of 2x Qiagen Multiplex PCR Master Mix (Qiagen) and 4 μl of sterile double-distilled water. PCR1 conditions were as follows: 95°C for 15 minutes followed by cycling (35x) conditions of 94°C for 30 seconds, 61°C for 90 seconds and 72°C for 60 seconds, and a final elongation step at 72°C for 10 minutes. Products were verified on a 1% agarose gel and cleaned using AmPure XP beads at a 1:1 ratio following the Agencourt AMPure XP PCR Purification Kit protocol (Beckman Coulter Genomics, Indianapolis) to remove primer dimers and nontarget DNA fragments. Purified products were resuspended in 15 μl of low TE. Following this, cleaned PCR1 products from the two MHC-I primer combinations (forward primers: HN36 and HN38, Additional file 1: Table S1) were combined. Second-round PCR (PCR2) amplification to add Illumina indexes was undertaken in a total volume of 20 µl and used 8 μl of template (separately for pooled products from PCR1 for MHC-I, and PCR1 products for MHC-II), 1 µl of Fi5 and Ri7 illumina indexes (2 µM), and 10 µl of 2x Qiagen Multiplex PCR Master Mix (Qiagen). PCR2 conditions were as follows: 95°C for 15 minutes followed by cycling (12x) conditions of 98°C for 10 seconds, 65°C for 30 seconds and 72°C for 30 seconds, and a final elongation step at 72°C for 10 minutes.

Peak distributions from products before and after PCR2 were checked using an Agilent Tapestation 4200 (Agilent Technologies) for a subset of 8 samples to confirm barcodes had been attached. Concentrations of all PCR2 products were then quantified using a QuantiFluor dsDNA kit (Promega) assay. Subsequently, MHC-I and MHC-II PCR2 products were separately pooled on an equimolar basis into groups of 24, resulting in a total of 26 pools, each pool equalised to 80 ng in 20 µl. Pooled amplicons were cleaned first with a 0.5 amplicon :1 ratio, and then again with a 1:1 ratio, of AmPure XP beads, and eluted with 20 μl of low TE. Each pool was quantified via qPCR (Applied Biosystems, California) using the KAPPA library quantification kit (Kappa Biosystem) and checked using a Qubit 4.0 Fluorometer (Invitrogen, Carlsbad) with a Qubit dsDNA BR assay kit (Invitrogen, Carlsbad) before being pooled to create two pools (MHC-I and MHC-II), each at 4 nM. Each final amplicon pool was purified, and size selected using Pippin prep. Final concentrations were quantified with qPCR, and correct peak distribution checked with the Agilent Tapestation 4200 (Agilent Technologies) before the MHC class I and II pools were combined at a 4 nM concentration for subsequent sequencing using 2x 250-bp paired-end sequencing on an Illumina MiSeq (Illumina, San Diego).

*Microbial sequencing*

Aliquots of gDNA were shipped on dry ice to the Centre for Genomic Research, University of Liverpool for library preparation, pooling and sequencing. Bacterial barcoding was performed with a 2-step amplification process using the primers 515F (5'TGCCAGCMGCCGCGGTAA3’) and 806R (5’GGACTACHVGGGTWTCTAAT3’) [70], which amplify the V4 region of the 16S rRNA gene. In brief, PCR1 amplification occurred in a total volume of 20 µl and used 5 ng gDNA, 0.5 µl of forward and reverse 16S V4 specific primers (10 µM), and 10 µl of 2x Kapa HiFi amplification mix. PCR1 conditions were as follows: 95°C for 2 minutes (hot start) followed by cycling (15x) conditions of 98°C for 20 seconds, 65°C for 15 seconds and 72°C for 30 seconds. Reactions were finished with a 72°C incubation for 5 minutes. PCR products were cleaned using ampure beads at a 1:1 ratio and resuspended in a total volume of 9 µl. PCR2 amplification occurred using 0.5 µl of the forward i7 and reverse i5 primers (as in MHC methods) using the same conditions as PCR1, but with 20 cycles. PCR2 products were again cleaned with a 1:1 ratio of ampure beads. Concentrations of each pool were quantified using a Qubit Fluorometer and the correct peak distribution was checked with the Agilent Bioanalyser DNA HS chip. PCR2 Products were then pooled on an equimolar basis. The amplicon pool was purified, and size selected by Pippin prep, then checked via qPCR before sequencing using 2 x 250-bp paired-end Illumina MiSeq sequencing (Illumina, San Diego).

**Table S1: Primer sequences used for MHC sequencing.** Degenerate bases are

shown according to IUPAC codes: Y = C/T, N = any base.

| **MHC class** | **Primer name** | **5’ to 3’ sequence** | **Direction** |
| --- | --- | --- | --- |
| **MHC-II exon 2** | **cO33** | CACCNCCTGACCTGTGTCC | F |
| **MHC-II exon 2** | **cO43** | CGAGGGGACAYGCTCTGCC | R |
| **MHC-I exon 3** | **HN36** | TCCCCACAGGTCTCCACACAGT | F |
| **MHC-I exon 3** | **HN38** | TCCCCACAGGTCTCCACACACG | F |
| **MHC-I exon 3** | **HN46** | ATCCCAAATTCCCACCCACCTT | R |

**Table S2: Repeatability of MHC-I (n = 26) and MHC-II (n = 24) genotyping for different dominant frequency thresholds.** Minimum amplicon frequency was kept constant at 0.3%. Threshold with the greatest repeatability is in bold.

| dominant frequency threshold (%) | MHC-I | | MHC-II | |
| --- | --- | --- | --- | --- |
|  | Repeatability (%) | average number of alleles | Repeatability (%) | average number of alleles |
| 5 | 77.83 | 10.17 | 78.63 | 29.00 |
| 10 | 77.67 | 10.13 | 84.38 | 34.42 |
| 15 | 77.48 | 8.79 | 85.58 | 36.31 |
| 20 | 77.89 | 7.85 | 86.09 | 36.52 |
| 25 (default) | **78.69** | 7.48 | **86.37** | 36.44 |

**Table S3: Repeatability of MHC-I (*n* = 26) and MHC-II (*n* = 24) genotyping for different minimum amplicon frequencies.** Minimum dominant frequency threshold was kept constant at 25%. Minimum amplicon frequency with the greatest repeatability for each MHC class is in bold.

| Minimum amplicon frequency (%) | MHC-I | | MHC-II | |
| --- | --- | --- | --- | --- |
|  | Repeatability (%) | average number of alleles | Repeatability (%) | average number of alleles |
| 0 | 70.79 | 13.85 | 91.09 | 58.94 |
| 0.1 | 70.74 | 10.58 | 88.12 | 54.19 |
| 0.2 | 74.97 | 8.96 | 87.99 | 42.81 |
| 0.3 | 78.69 | 7.48 | 86.37 | 36.44 |
| 0.4 | 83.54 | 6.77 | 81.94 | 30.52 |
| 0.5 | 86.45 | 6.31 | 82.03 | 25.27 |
| 0.6 | 87.01 | 6.13 | 84.00 | 21.31 |
| 0.7 | 90.93 | 5.79 | 84.01 | 18.00 |
| 0.8 | 91.58 | 5.71 | 85.05 | 15.88 |
| 0.9 | 91.46 | 5.52 | 84.19 | 14.10 |
| 1 | 90.9 | 5.44 | 83.93 | 12.46 |
| 1.5 | 94.11 | 5.06 | 87.18 | 7.71 |
| 1.6 | **95.48** | **4.96** | 89.93 | 6.96 |
| 1.7 | 94.78 | 4.92 | 88.87 | 6.73 |
| 1.8 | 93.03 | 4.87 | **90.12** | **13.04** |
| 1.9 | 92.66 | 4.90 | 89.70 | 12.79 |
| 2 | 93.34 | 4.75 | 87.80 | 6.02 |
| 3.00 (default) | 90.3 | 4.13 | 79.89 | 3.50 |

**Table S5:** **Core families present in 281 faecal samples, collected from 224 Seychelles warblers.** Core microbiome is defined as bacterial families that appeared in at least 50% of samples, with a minimum relative abundance of 0.1%. Total number of reads, and % of all reads are included.

**Table S5: The effect of host-associated variables on gut microbiome diversity in the Seychelles warbler (n = 195).** GLMMs for three metrics of alpha diversity: Shannon diversity, Chao 1(log transformed) and Faiths phylogenetic diversity (log transformed): **A**) including the presence/absence of MHC alleles or, **B**) MHC diversity. A Linear model was used to generate conditional model-averaged estimates (β), their standard error (SE), z value, *P* value, and relative importance (ω) are shown for all predictors featuring in the top model set (ΔAIC_c_ ≤ 7). All continuous factors were standardised. Estimates are in reference to MHC allele = absent, *TLR3* genotype = *TLR3*^AA^, sex = female, age class = fledgling, field period = Major 2017. Significant terms are in bold and underlined. *** *P* < 0.001, ** *P* < 0.01, * *P* < 0.05.

**
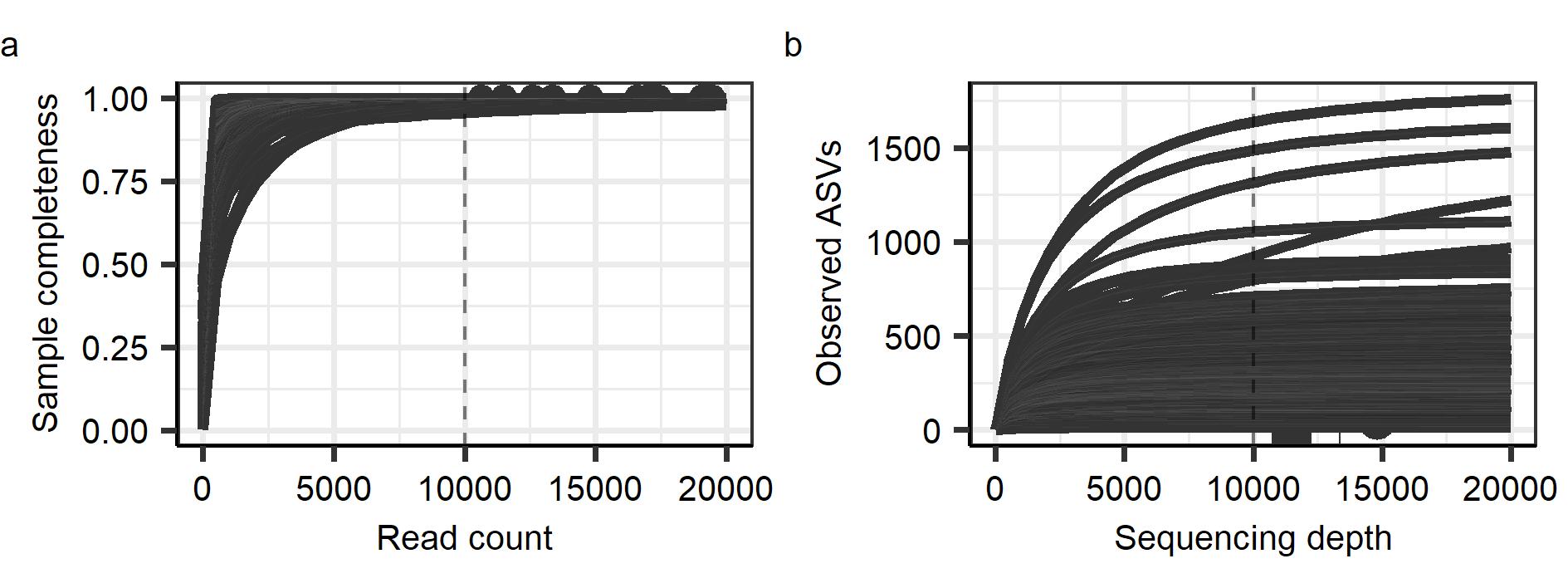
**

**Fig S1**: **(A) Sample completeness and (B) rarefaction curves** **in Seychelles warbler faecal samples.** Each line represents a single faecal sample (281 faecal samples, collected from 224 Seychelles warblers). Curves were generated using the R package iNEXT 2.0.20, with 50 bootstrap replicates per sample. The dashed line represents the number of reads used as a cut-off for retaining samples in downstream analysis (all samples with fewer than 10,000 reads were removed).


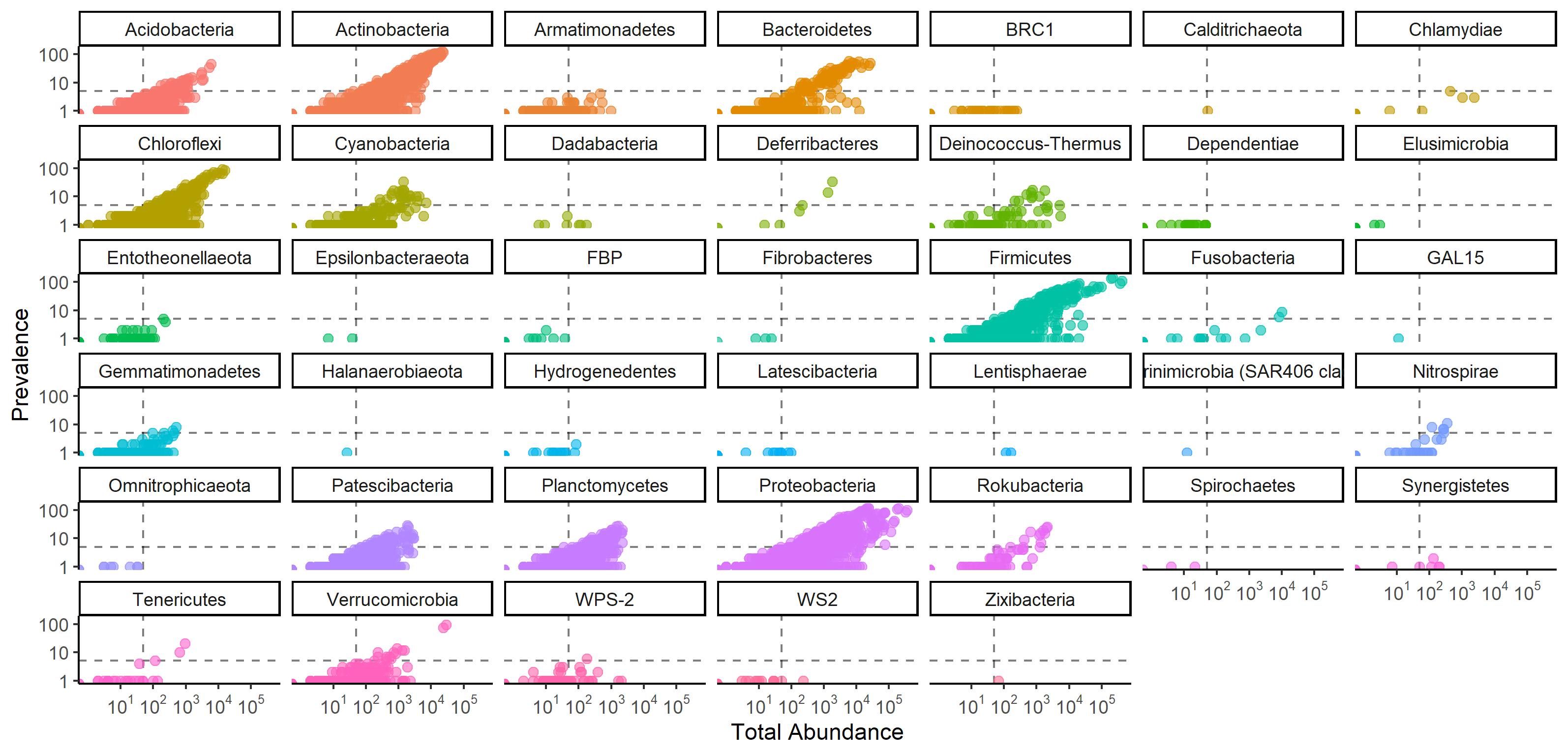


**Fig S2: Prevalence and total abundance of all ASV’s separated by phylum**. Each phylum is shown in a separate plot, and a different colour. Dashed lines represent the values used as cut-offs for filtering rare taxa before alpha and beta diversity analyses (minimum abundance = 50), and additional filtering for beta diversity (prevalence threshold = 2.5%).

**
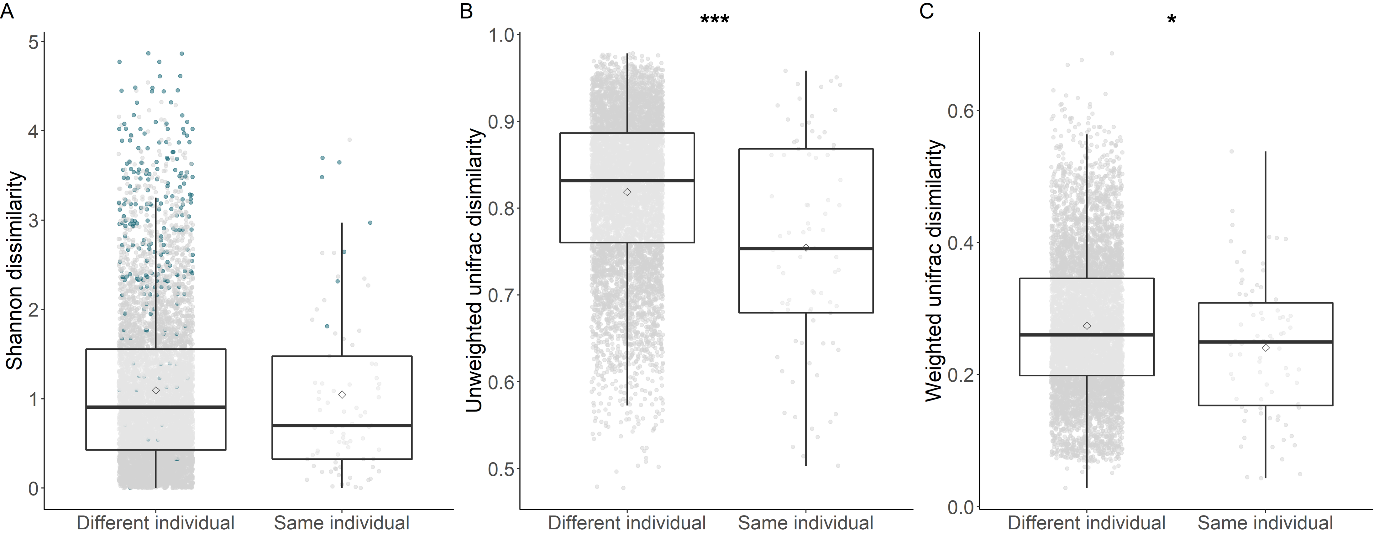
**

**Fig S3:** **Individual repeatability of alpha and beta diversity measures in the Seychelles warbler.** This was tested by sequencing multiple samples taken from the same individuals; these samples were collected during the same field season (n = 115 faecal samples from 51 individuals. Pairwise Euclidean distances were calculated between samples taken from different individuals, versus those from within the same individual, in the same season for **A**). Shannon dissimilarity **B**) unweighted UniFrac dissimilarity and **C**) weighted UniFrac dissimilarity. Boxes span the interquartile (25% - 75%) range. Whiskers extend to 1.5 times the interquartile range. The median is marked by a horizontal line and the mean is marked by a diamond. Dark blue points in **A)** indicate pairwise comparisons involving two outliers. Significant differences are shown, and *P*-values are derived from Kruskal–Wallis tests: *** *P* < 0.001, * *P* < 0.05.

**
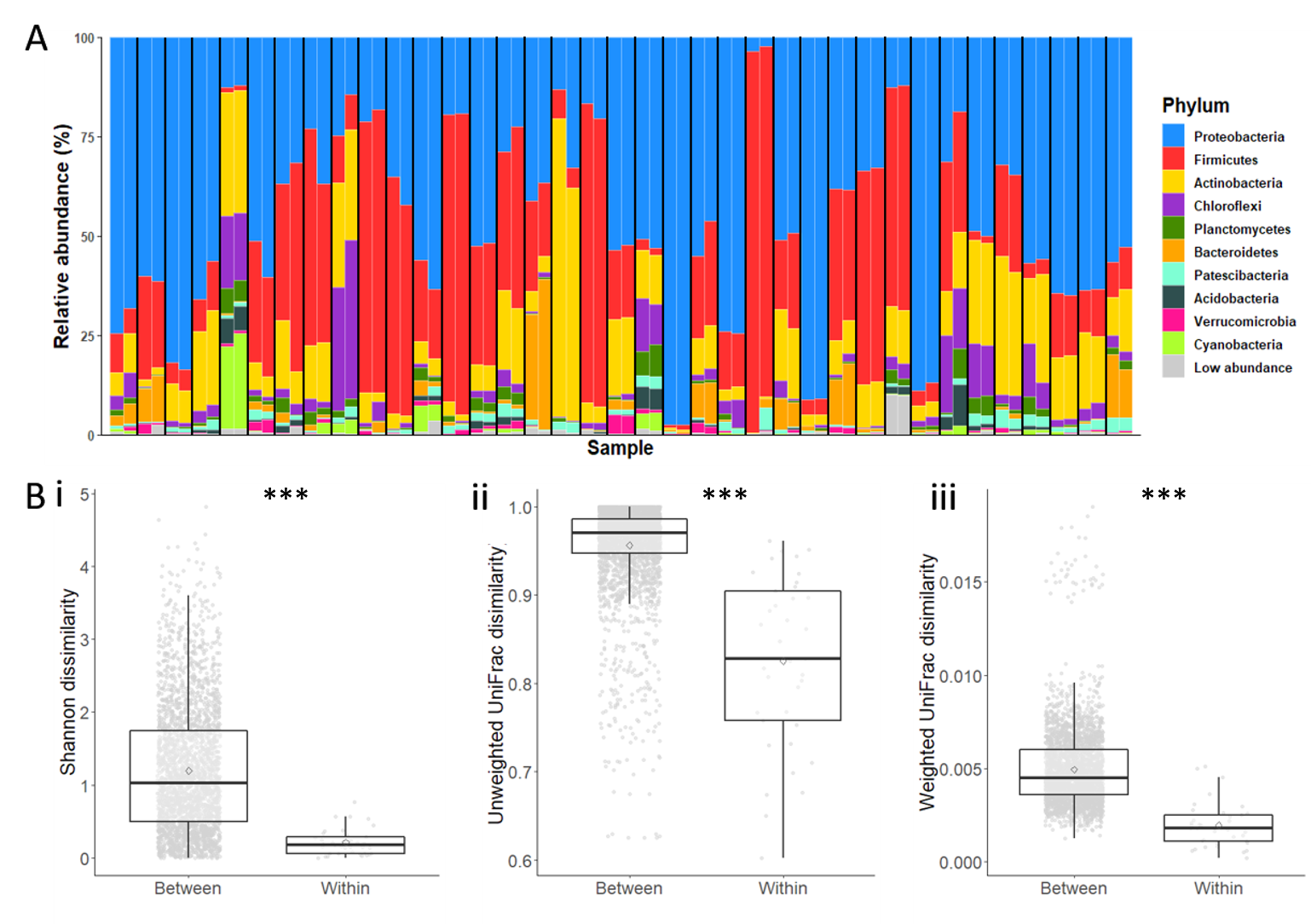
**

**Fig S4:** **The repeatability of sequencing methods.** This was tested by sequencing 37 faecal samples taken from individual Seychelles warblers twice. **A**) Relative abundance (%) of the 10 most abundant taxa at the phylum level for the 37 duplicated samples. Each column represents one sample, black lines separate duplicated samples. All other taxa within each sample are collapsed into the low abundance category. **B**) The pairwise Euclidean dissimilarity between different samples, versus within pairs of duplicated samples (same DNA sequenced twice) for **i**. Shannon dissimilarity **ii**. unweighted UniFrac dissimilarity and **iii**. weighted UniFrac dissimilarity. Boxes span the interquartile (25% - 75%) range. Whiskers extend to 1.5 times the interquartile range. The median is marked by a horizontal line and the mean is marked by a diamond. Significant differences are shown, and P-values were derived from Kruskal–Wallis tests: *** *P* < 0.001.


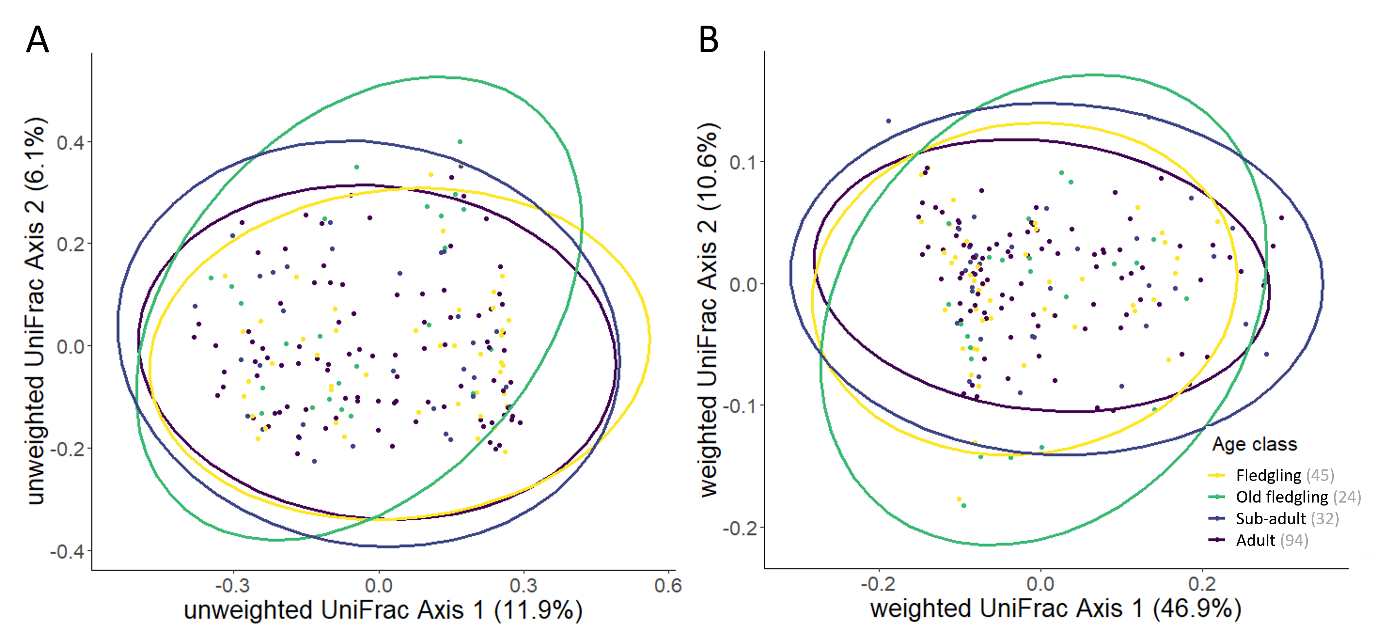


**Fig S5**: **Beta diversity of Seychelles warbler gut microbiome composition in different age classes.** The principal coordinate plots are based on **A)** unweighted UniFrac distances, and **B)** weighted UniFrac distances. Points represent a single faecal sample from a different individual (*n* = 195). Sample sizes are specified in brackets in the legend, and colours indicate the age class which was either fledgling (yellow), old-fledgling (green), sub-adult (indigo) and adult (purple). Ellipses represent a 95% confidence interval around the cluster centroids.


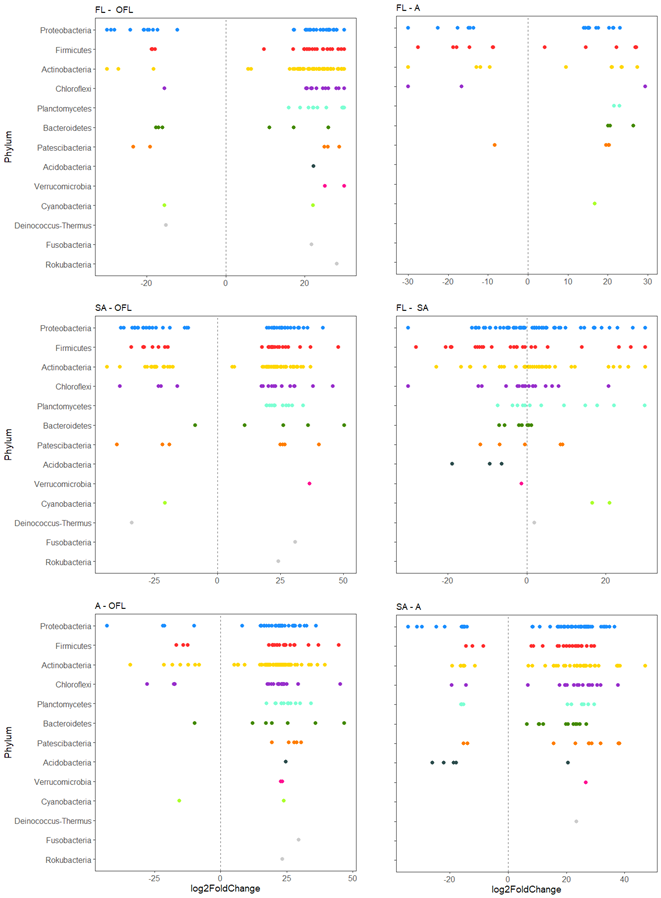


**Fig S6**: Differentially abundant ASV’s in the gut microbiome of Seychelles warblers between different age categories (FL = fledgling, OFL = old fledgling, SA = sub-adult, A = adult). ASVs are grouped at the level of bacterial order and coloured according to bacterial phylum. Differential ASV abundance was assessed using negative binomial Wald tests and *P* values were adjusted using the Benjamini and Hochberg false-discovery rate correction with a significance cut-off of *P* < 0.01. ASVs shown with a log_2_ fold change greater than zero are significantly more abundant in the age classes on the left and ASVs with a log_2_ fold change smaller than zero are significantly more abundant in age classes on the right.


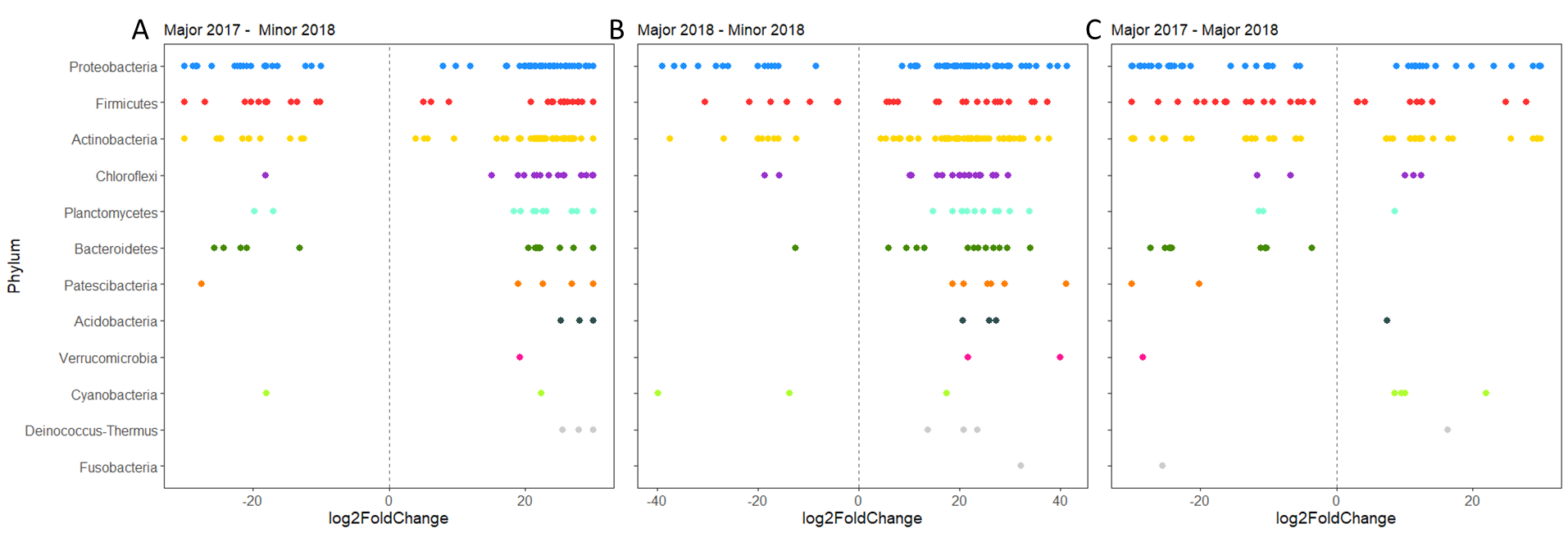


**Fig S7**: Differentially abundant ASV’s in the gut microbiome of Seychelles warblers, between seasons. Comparisons are **A)** Major 2017 vs Minor 2018, **B)** Major 2018 vs Minor 2017, or **C)** Major 2017 vs Major 2018. ASVs are grouped at the level of bacterial order and coloured according to bacterial phylum. Differential ASV abundance was assessed using negative binomial Wald tests and *P* values were adjusted using the Benjamini and Hochberg false-discovery rate correction with a significance cut-off of *P* < 0.01. ASVs shown with a log_2_ fold change greater than zero are significantly more abundant in seasons on the left and ASVs with a log_2_ fold change smaller than zero are significantly more abundant in seasons on the right.
